# Nanoparticle exposure reactivates latent herpesvirus and restores a signature of acute infection

**DOI:** 10.1186/s12989-016-0181-1

**Published:** 2017-01-10

**Authors:** Christine Sattler, Franco Moritz, Shanze Chen, Beatrix Steer, David Kutschke, Martin Irmler, Johannes Beckers, Oliver Eickelberg, Philippe Schmitt-Kopplin, Heiko Adler, Tobias Stoeger

**Affiliations:** 1 Helmholtz Zentrum München - German Research Center for Environmental Health (GmbH), Comprehensive Pneumology Center, Institute of Lung Biology and Disease, Ingolstädter Landstr. 1, D-85764 Neuherberg, Germany; 2 Helmholtz Zentrum München - German Research Center for Environmental Health (GmbH), Research Unit BioGeoChemistry, Ingolstädter Landstr. 1, D-85764 Neuherberg, Germany; 3 Comprehensive Pneumology Center, Research Unit Lung Repair and Regeneration, Helmholtz Zentrum München - German Research Center for Environmental Health (GmbH), Marchioninistrasse 25, D-81377 Munich, Germany; 4 University Hospital Grosshadern, Ludwig-Maximilians-University, D-81377 Munich, Germany; 5 Comprehensive Pneumology Center, Member of the German Center of Lung Research (DZL), D-81377 Munich, Germany; 6 Helmholtz Zentrum München - German Research Center for Environmental Health (GmbH), Institute of Experimental Genetics, Ingolstädter Landstr. 1, D-85764 Neuherberg, Germany; 7 German Center for Diabetes Research (DZD), Ingolstädter Landstr. 1, D-85764 Neuherberg, Germany; 8 Technische Universität München, Chair of Experimental Genetics, D-85354 Freising, Germany

**Keywords:** Carbonaceous nanoparticles (CNP), Double-walled carbon nanotubes (DWCNT), Intratracheal instillation, Persistent virus infection, Virus reactivation, Phospholipids

## Abstract

**Background:**

Inhalation of environmental (nano) particles (NP) as well as persistent herpesvirus-infection are potentially associated with chronic lung disease and as both are omnipresent in human society a coincidence of these two factors is highly likely. We hypothesized that NP-exposure of persistently herpesvirus-infected cells as a second hit might disrupt immune control of viral latency, provoke reactivation of latent virus and eventually lead to an inflammatory response and tissue damage.

**Results:**

To test this hypothesis, we applied different NP to cells or mice latently infected with murine gammaherpesvirus 68 (MHV-68) which provides a small animal model for the study of gammaherpesvirus-pathogenesis in vitro and in vivo. In vitro, NP-exposure induced expression of the typically lytic viral gene ORF50 and production of lytic virus. In vivo, lytic viral proteins in the lung increased after intratracheal instillation with NP and elevated expression of the viral gene ORF50 could be detected in cells from bronchoalveolar lavage. Gene expression and metabolome analysis of whole lung tissue revealed patterns with striking similarities to acute infection. Likewise, NP-exposure of human cells latently infected with Epstein-Barr-Virus also induced virus production.

**Conclusions:**

Our results indicate that NP-exposure of persistently herpesvirus-infected cells – murine or human – restores molecular signatures found in acute virus infection, boosts production of lytic viral proteins, and induces an inflammatory response in the lung – a combination which might finally result in tissue damage and pathological alterations.

**Electronic supplementary material:**

The online version of this article (doi:10.1186/s12989-016-0181-1) contains supplementary material, which is available to authorized users.

## Background

The rapid expansion of nanotechnology is expected to bring considerable benefit to mankind, but at the same time, newly developed materials might pose new and unknown risks to exposed people. Inhalation of high levels of spherical carbonaceous nanoparticles (CNP) – surrogates for combustion derived nanoparticles – has been shown to induce an inflammatory phenotype in the lungs of healthy mice [1] as well as acute extra-pulmonary cardiovascular distress [2]. Comparing a panel of different CNPs revealed particle surface related oxidative stress to be the common driver of the acute response to particles of low solubility and low toxicity [3, 4]. At moderate doses, CNP-triggered acute inflammation has been shown to resolve within several days after exposure in noncompromised mice [5]. Yet, repeated inflammatory events or exposure of individuals with higher susceptibility to NP-associated adverse effects, such as asthmatics [6], might provoke severe damage to the lung tissue. Recent research indicates that at equal surface dose, fiber shaped carbon nanotubes (CNT) – which due to their rapidly increasing mass production gain increasingly more environmental importance – also generate an acute inflammatory response via oxidative stress pathways, but in contrast to spherical particles, the CNT-induced pulmonary inflammation persists over weeks [7]. Inhaled nanoparticles generally deposit efficiently and persistent in the respiratory tract, and may due to their pro-inflammatory potency represent one environmental factor contributing to the development of lung diseases including asthma, chronic obstructive lung disease (COPD) and potentially even interstitial pulmonary fibrosis or cancer [8]. An additional environmental factor driving the susceptibility for chronic lung disease could be virus infection. A number of studies imply that especially herpesviruses might contribute to the development of lung diseases such as idiopathic pulmonary fibrosis (IPF). In lungs of patients affected from IPF, DNA of one, two or even more herpesviruses has been detected by PCR, suggesting an association between chronic virus infection and IPF [9, 10]. In particular, proteins and DNA from Human Cytomegalovirus (HCMV) and Epstein-Barr-Virus (EBV) have frequently been detected. Due to the species-specificity of HCMV and EBV, pathogenic studies of the human infections are restricted. Thus, animal models are needed and the murine gammaherpesvirus 68 (MHV-68) provides such an animal model [11, 12]. MHV-68 has been shown to act as a cofactor in bleomycin-induced fibrosis [13, 14] and to exacerbate fluorescein isothiocyanate-induced pulmonary fibrosis [14, 15]. Furthermore, infection of Th2-biased mice with MHV-68 induces the development of progressive lung fibrosis with pathological features also seen in IPF [16]. Upregulation of profibrotic or proinflammatory factors in infected cells and repeated virus reactivation followed by lytic replication events are supposed to be important factors in the development or exacerbation of IPF in these models [17, 18]. Control of viral latency and prevention of productive virus replication depends on a highly complex balance between immune surveillance and regulation of viral and cellular gene expression [12, 19, 20]. Both the immune response and the metabolism are important players in surveillance of viral latency and regulation of immune responses within this context. Viruses have been shown to alter metabolic pathways of their host cells in a highly specific manner to generate optimal conditions not only for virus replication and production of new virus particles but also for maintenance of viral latency [21, 22]. Simultaneously, immune responses and metabolism are increasingly considered to be closely linked, e.g. by sharing pathways and being crossregulated [23, 24], suggesting a well-established balance between metabolic alterations caused by the virus and modifications due to counteractions by the host. We hypothesize that inhalation of NP as a second hit disrupts this balance and interferes with the ability to control viral infection, which might finally result in a non-resolving, chronic inflammation and even fibrosis. At present, no information about the health relevance of a suchlike liaison of NP and persistent virus infection is available.

In this study, we show that the presence of NP induces the production of lytic virus from persistently infected murine cells in vitro. In vivo, exposure to NP by intratracheal instillation leads to an increase in the expression of lytic viral proteins in lungs of mice persistently infected with MHV-68 and creates transcriptome and metabolome signatures in the lung with considerable parallels to the ones observed during the acute phase of virus infection. NP exposure of human cells that are latently infected with EBV also induces reactivation of latent virus, indicating that the NP-effect is not limited to the murine system. Taken together, our results suggest that the combination of persistent herpesvirus infection and NP exposure disrupts the immune control of viral latency by altering cellular metabolism and gene expression.

## Results

### NP exposure in vitro boosts lytic virus replication in pseudo-latently infected cells

**Fig. 1:**
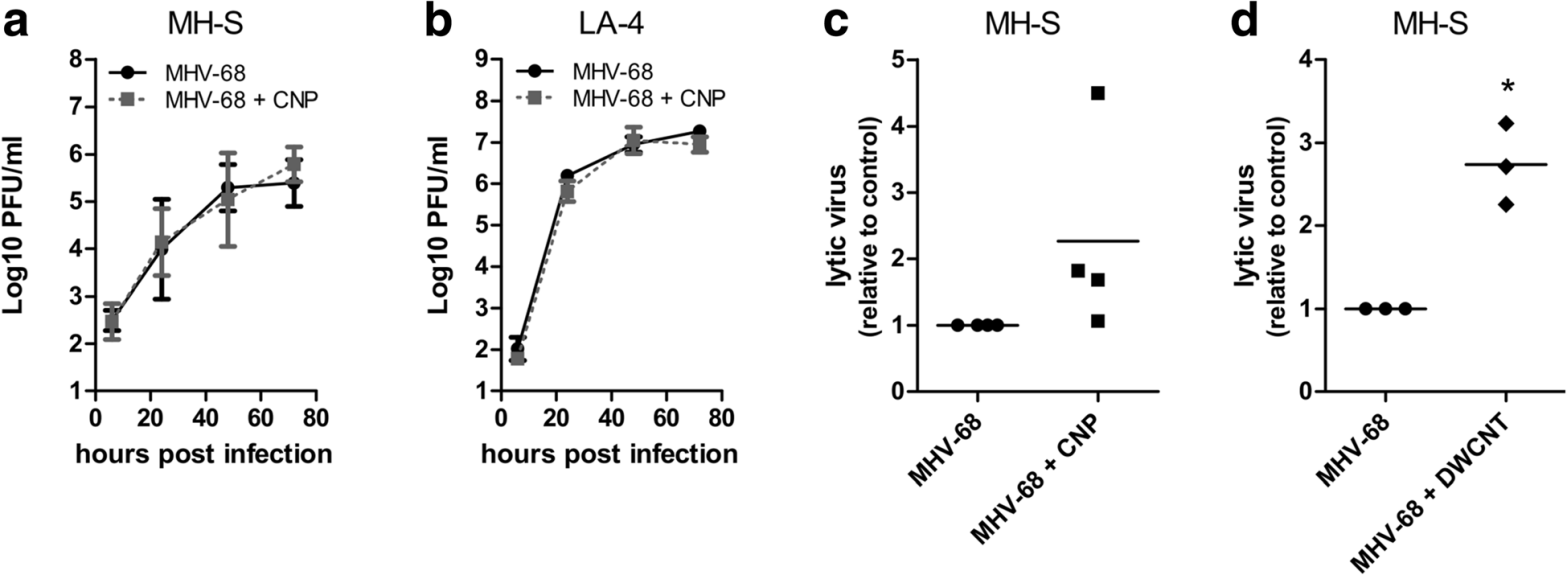
Exposure to NP does not alter lytic virus replication after primary infection in vitro but boosts lytic replication in persistently infected cells: The alveolar macrophage cell line MH-S **a** or the alveolar epithelial cell line LA-4 **b** were infected for 2 h with MHV-68 wildtype virus at an MOI of 1 and then treated with 50 μg/ml CNP. Cells and cell culture supernatants were harvested at different time points post infection, and titers were determined by plaque assay on BHK-21 cells. Data shown are the means ± SD from two independent experiments. **c** and **d**: MH-S cells were infected with MHV-68 overnight, treated with 50 μg/ml CNP **c** or DWCNT **d** and incubated for another two hours. Free lytic virus was inactivated by incubation with citrate buffer (pH = 3.0). After plating of serial cell dilutions on indicator cells, the amount of cytopathic effect (CPE) was determined. Relative values, normalized to the genomic load in the infected cells, were calculated. The value for untreated cells was set as “1”. Symbols represent values from individual experiments and the bars represent the mean. Asterisks indicate a statistically significant difference to the untreated control (*: *P* < 0.05)

MHV-68 such as all herpesviruses is characterized by its complex life cycle consisting of an acute lytic infection and a lifelong maintained latent infection. In this study, we wanted to analyze the influence of NP on both phases of the viral life cycle. To investigate lytic virus growth after primary infection in the presence or absence of NP, we performed multistep growth curves in murine alveolar epithelial cells (LA-4 cell line) and alveolar macrophages (MH-S cell line). Alveolar macrophages act as the first defense against inhaled NP [25] whereas alveolar epithelial cells create the large respiratory surface area of the lung and are known to support acute lytic virus replication after intranasal infection [12]. As stimulating carbonaceous NP, we used Printex 90 (CNP) which represents a widely used and well characterized type of carbon black with well described effects on inflammatory and oxidative stress related processes [26, 27]. We used doses of 50 μg/ml as aqueous dispersions with an averaged agglomerate diameter (Z-Ave) in medium of 337 ± 5 nm (see also Additional file 1: Fig. S1), which did not reduce cell viability to less than 80% on average neither in MH-S nor in LA-4 cells, as tested by WST-assay (Additional file 1: Fig. S2a and b). Both cell lines supported lytic virus growth and virus titers increased by 3 (MH-S cells) to 5 logs (LA-4 cells) over a period of 96 h (Fig. 1a and b). However, no changes in virus titers in the presence of CNP could be observed. Therefore, we suppose that CNP do not affect lytic virus growth in de novo infected cells. In the next step, we established a cell culture assay to detect low-level lytic virus replication in pseudo-latently infected cells by a slight modification of a previously published method [28] to evaluate if CNP can boost low-level lytic virus replication, an event that can be observed in latently infected individuals at regular intervals [12]. As macrophages have been shown to harbor latent MHV-68 [29], we again used MH-S cells as target cells in these experiments. MH-S cells were first infected with MHV-68 at a low multiplicity of infection (MOI = 0.01) and then treated with NP. Remaining extracellular lytic virus was inactivated and serial dilutions of cells were plated on indicator cells to study the development of a cytopathic effect (CPE). Control assays performed with cells that were mechanically disrupted by two freeze-thaw cycles before plating yielded an insignificant amount of CPE, indicating that the lytic virus detected in this assay was actively produced by living MH-S cells and not due to remaining input virus. Exposure of infected cells to CNP increased the amount of lytic virus in three out of four experiments (Fig. 1c). To test if this observation was specific for the type of carbonaceous NP, we also analyzed the effect of fiber-shaped double-walled carbon nanotubes (DWCNT). Addition of DWCNT dispersions (Z-Average of 26 ± 1 nm; see also Additional file 1: Fig. S1) to the cells, at a dose of 50 μg/ml, induced significantly higher amounts of lytic virus when compared to untreated controls (Fig. 1d). The used concentration of DWCNT showed some impact on cell survival and reduced cell viability by roughly 30% (Additional file 1: Fig. S2b). Our results demonstrate that exposure to NP boosts the release of lytic virus from pseudo-latently infected cells irrespective of the type of NP.

### NP exposure of persistently infected cells reactivates latent virus

**Fig. 2:**
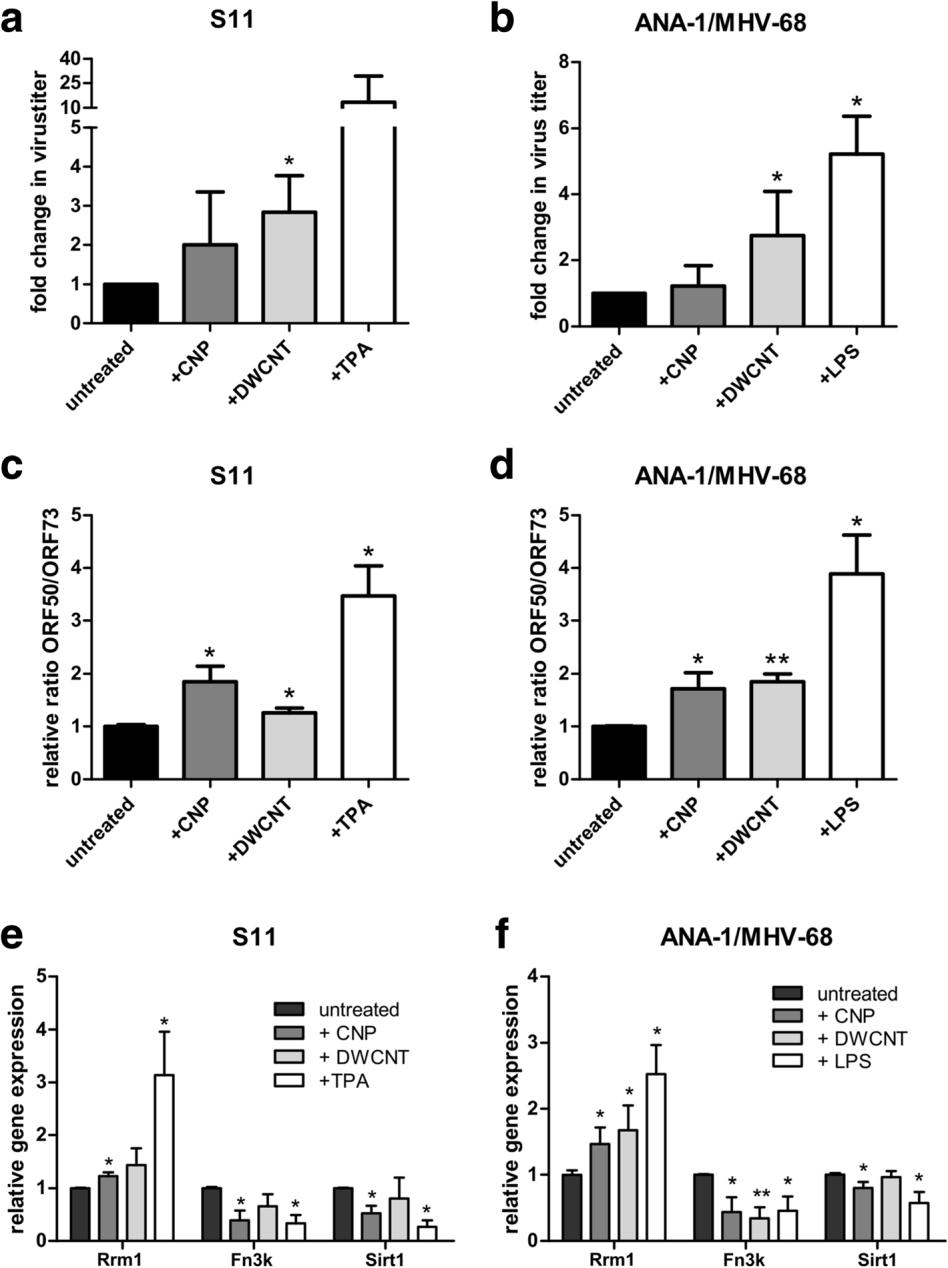
Exposure to NP reactivates latent virus in persistently infected cells in vitro: The S11 B-cell line and the persistently with MHV-68 infected macrophage cell line ANA-1/MHV-68 were incubated with 50 μg/ml NP and lytic virus was determined in the supernatant by plaque assay after 72 h (panels **a** and **b**). Expression of the viral genes ORF50 (specific for the lytic phase) and ORF73 (expressed during lytic and latent phase) – shown as the ratio ORF50/ORF73 - (panels **c** and **d**) and of genes that have been shown to be associated with reactivation of latent virus (panels **e** and **f**) was analyzed by RT-PCR 72 h after NP exposure in S11 and in ANA-1/MHV-68 cells. The values in untreated cells were set as “1” and the values for cells after NP treatment were calculated relative to the control. Treatment with TPA or LPS, respectively, was used as a positive control. Data shown are the means + SD from three (S11 cells; panels **a**, **c**, **e**) or four independent experiments (ANA-1/MHV-68 cells; panels **b**, **d**, **f**). Asterisks indicate a statistically significant difference to the untreated control (*: *P* < 0.05; **: *P* < 0.01)

Since the described system used with MH-S cells is not a true “latency-model”, we performed additional experiments with two latently infected cell lines to investigate if NP-exposure can actually reactivate latent virus. First, the B cell line S11 was used, which has been isolated from the spleen of a mouse with MHV-68-associated lymphoproliferative disease [30]. Second, we established a persistently infected macrophage cell line by infecting the bone marrow-derived cell line ANA-1 with a recombinant virus carrying a hygromycin-resistance as described in materials and methods (= ANA-1/MHV-68). The establishment of a latently infected MH-S cell line by this method was technically not feasible, probably due to the fact that MH-S cells support lytic virus replication to a too high extent (see also results in Fig. 1a). We incubated both cell lines with three different doses of NP (5 μg/ml, 20 μg/ml and 50 μg/ml) to determine the optimal dose. Lytic virus was determined in the supernatant by plaque assay after 72 h (Additional file 1: Fig. S3a and b). Treatment with 12-O-tetradecanoyl-phorbol-13-acetate (TPA) was used as a positive control for the induction of virus reactivation in S11 B cells [31], and treatment with lipopolysaccharide (LPS) in ANA-1/MHV-68 macrophages [32]. Exposure to 50 μg/ml NP resulted in an increase in virus titers in the supernatants of both cell lines, indicating virus reactivation. At this dose, both NP showed a slight effect on cell survival and reduced viability by 11% (CNP) or 29% (DWCNT) in uninfected ANA-1 cells (Additional file 1: Fig. S2c). A dose of 50 μg/ml NP was used in the following experiments. S11 and ANA-1/MHV-68 cells were exposed to NP for 72 h and virus titers in the supernatant were determined (Fig. 2a and b). An increase of virus titers could be observed after exposure to both CNP and DWCNT, but particularly in ANA-1/MHV-68 cells DWCNT had a bigger impact than CNP. As DWCNT have a two times higher surface area compared to CNP, we also tested a surface-adapted amount of CNP in further experiments (100 μg/ml), but the effect of DWCNT was still higher than the one induced by CNP (Additional file 1: Fig. S4). We further analyzed the expression of the viral genes ORF50 and ORF73 72 h after NP exposure by RT-PCR (Fig. 2c and d and Additional file 1: Fig. S2c and d). Expression of ORF73 served for normalization since it is expressed throughout all phases of the viral life cycle [33]. ORF50, also referred to as the replication and transcription activator Rta, is exclusively expressed during lytic virus replication [33]. Thus, an increase in the relative ratio of ORF50/ORF73 indicates that lytic virus production is induced. Consistent with the finding of higher virus titers in the supernatants of cells after NP-exposure, the relative ratio of ORF50/ORF73 was increased in NP-exposed cells, indicating that ORF50 expression and thereby lytic virus replication is induced. A dose-dependency could be observed to a certain extent at least in ANA-1/MHV-68 cells treated with DWCNT and S11 cells treated with CNP (Additional file 1: Fig. S3c and d). A number of cellular genes have been described to be potentially involved in virus reactivation and therefore we analyzed if some of these genes are regulated by exposure of latently infected cells to NP. Five genes were chosen for further investigation by RT-PCR 72 h after exposure of S11 or ANA-1/MHV-68 cells to NP. Ribonucleoside-diphosphate reductase large subunit (Rrm1) is described as being upregulated in the context of reactivation of the human gammaherpesviruses EBV and Kaposi’s sarcoma-associated herpesvirus (KSHV) [34] while Fructosamine-3-kinase (Fn3k), Tousled-like Kinase 1 (Tlk1), TGFbeta-activated kinase 1/MAP3K7-binding protein 1 (Tab1), and Sirtuin 1 (Sirt1) have been found to be downregulated [35, 36]. Consistent with reactivation of latent virus, Rrm1, Fn3k and Sirt1 were up- or downregulated as described for EBV and KSHV, albeit to a variable degree depending on the cell line and the type of NP (Fig. 2e and f). Tab1 and Tlk1 proved to be non-regulated in both cell lines.

In summary, our in vitro results show that exposure of persistently infected cell lines to carbonaceous NP induces virus reactivation and alters the expression of cellular genes associated with virus reactivation independently of the NP’s type or aspect ratio, whereas acute virus growth in cells after de novo infection is not affected.

### Treatment of latently infected mice with NP induces the expression of lytic virus proteins in the lung

**Fig. 3:**
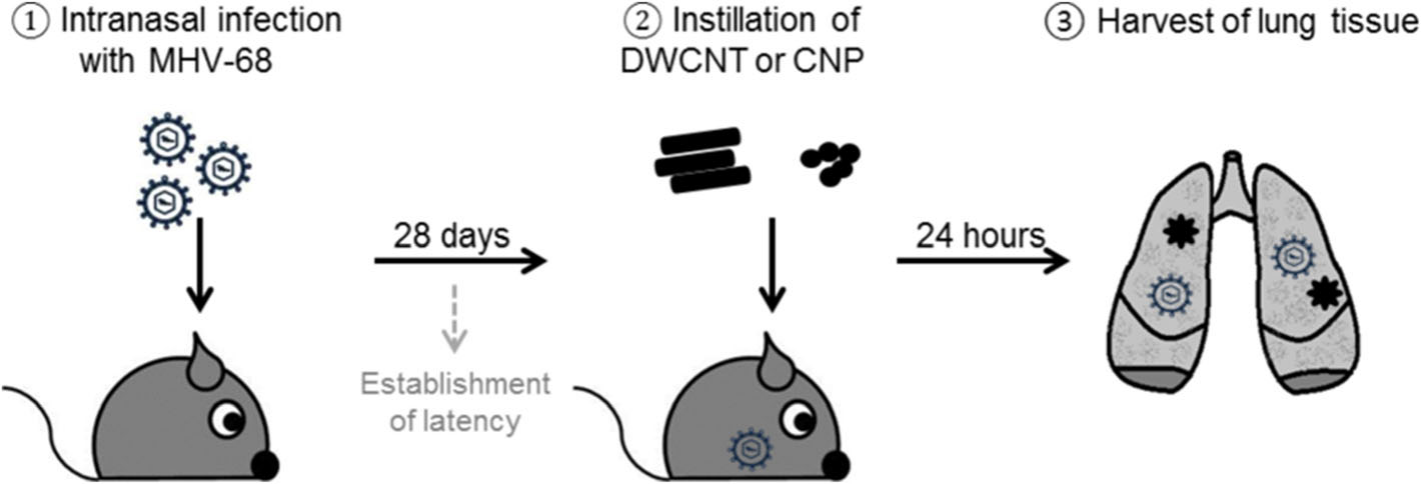
Schematic overview of the experimental setup in vivo: C57BL/6 mice were infected intranasally with 5 × 10^4^ PFU of MHV-68 (①). At day 28 post infection, when virus latency is established, the mice were instilled with 50 μg in 50 μl volume of spherical carbon nanoparticles (CNP) or double-walled fiber shaped carbon nanoparticles (DWCNT) after non-surgical intubation (②). Tissue samples of the lung were harvested 24 h after instillation for analysis of the metabolome, transcriptome, histology and viral load (③)

**Fig. 4:**
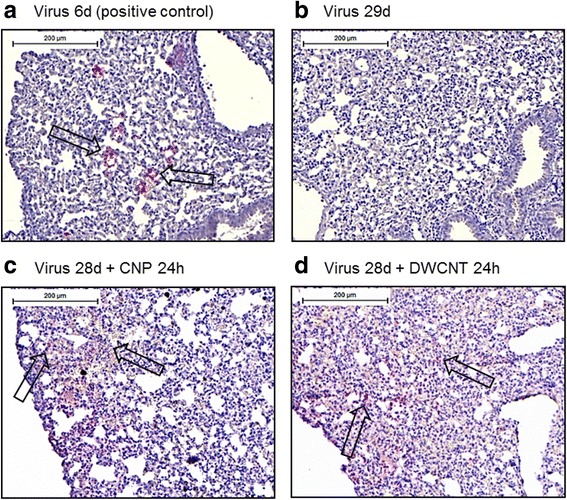
Immunohistochemical staining shows increased expression of lytic viral proteins in lungs persistently infected with MHV-68 and subsequently exposed to NP: Lung sections were stained with a rabbit-polyclonal serum directed against lytic proteins of MHV-68. Arrows indicate positive staining in the positive control **a** and in latently infected mice treated subsequently with NP **c** and **d**. No positive staining was detected in mice with latent virus only **b**. Scale bar: 200 μm. Representative stainings from 3 mice per group are shown

To test our in vitro findings also in vivo, we infected mice i.n. with MHV-68 (experimental setup see Fig. 3). At day 28 post infection – at the time when latent infection is known to be established in B cells, pulmonary epithelial cells and macrophages and lytic virus is no longer detectable [29, 37] – the mice were instilled intratracheally either with CNP or DWCNT or left untreated. Tissue samples of the lung were harvested for analysis 24 h later. A dose of 50 μg per mouse was chosen, since such a dimension of pulmonary carbon deposition could be achieved at maximal occupational settings (5 day and 8 h per day exposure at a carbon black occupational exposure limit of 3.5 mg/m^3^ [38], with a clearance of less than 3% per day for inhaled NP [39]). Lung sections were stained with a polyclonal serum against lytic proteins of MHV-68 and expression of lytic virus proteins could be detected in latently infected mice treated with NP (Fig. 4c and d) but not in mice with latent virus only (Fig. 4b). Similar to the acute infection situation, the presence of lytic virus seemed to be locally restricted, albeit it did not reach the dimensions observed in acute virus infection (Fig. 4a).

**Fig. 5:**
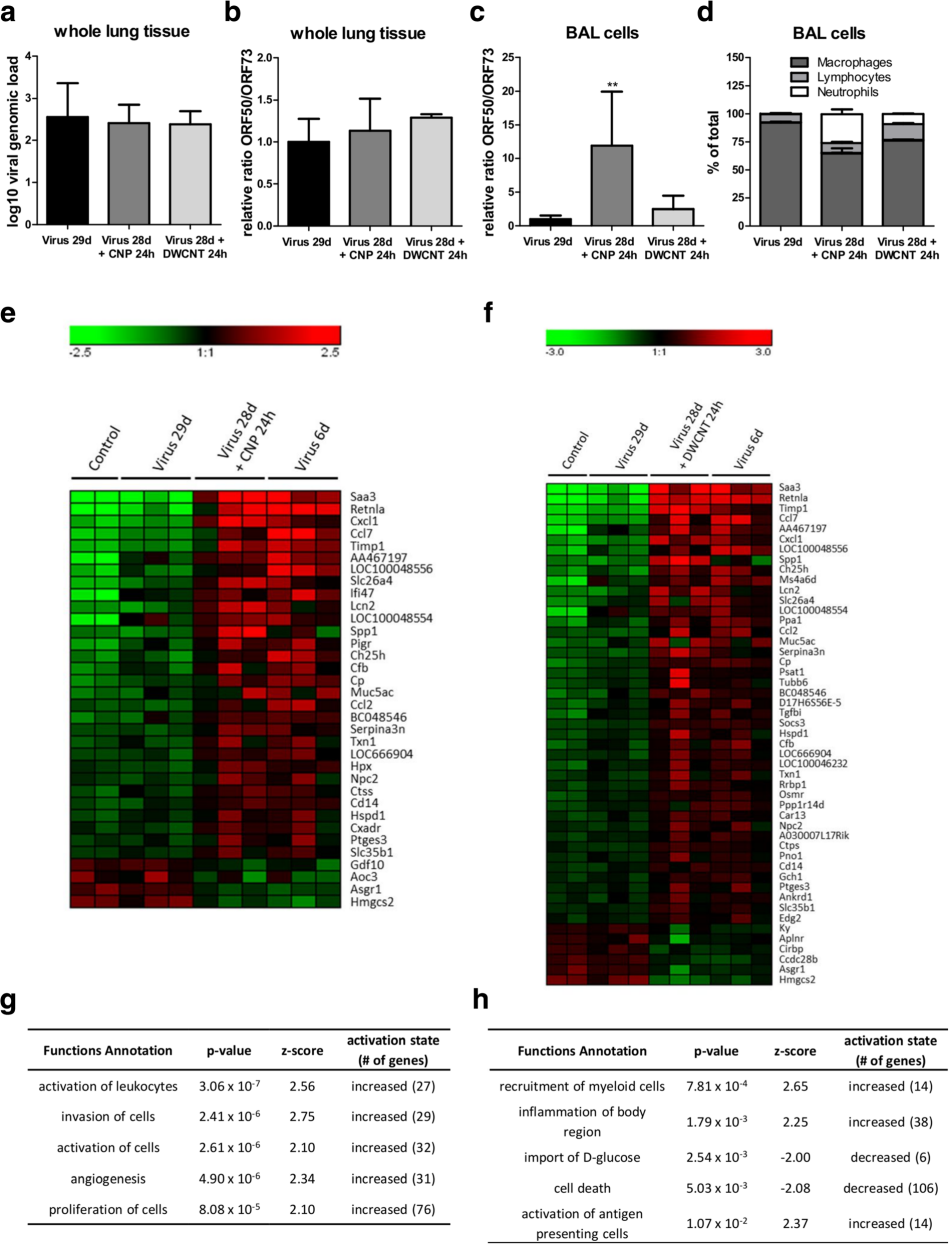
Treatment of latently infected mice with NP restores features resembling the ones seen in acute infection: The viral genomic load in whole lung tissue was measured by qPCR **a**. The expression of the viral genes ORF50 (specific for lytic replication) and ORF73 (expressed throughout all phases of the viral life cycle) was analyzed in whole lung tissue **b** and in BAL cells **c** by RT-PCR. The mean ratio between ORF50 and ORF73 expression detected in mice with virus for 29d only was set as “1” and all other values were expressed as relative values. Differential cell counts were made to examine the composition of BAL cells **d**. The transcriptome of whole lung tissue was analyzed with the Illumina-MouseRef-8v2.0 Expression BeadChip. Genes that were both regulated in the acute infection situation and in latent infection plus NP treatment compared to untreated control mice or to mice with latent virus or NP only were searched for in the transcriptome data. Only genes that were at least 1.5-fold up- or downregulated compared to all control groups were considered as differentially regulated. An overlap of 15 upregulated and 3 downregulated genes was found in the group subsequently treated with CNP **e**, and 33 upregulated plus 5 downregulated genes in the group treated with DWCNT **f**. Regulated pathways that were identified by IPA are shown in **g** for CNP and in **h** for DWCNT as a second hit in latently infected mice. All data were obtained from a single experiment with 6 mice per group for BAL cells and 3 mice per group for whole lung tissue, except for the control group in panels e and f, where the expression values of 2 mice are shown. In panels a, b, c and d, the means + SD are shown

We further analyzed if this induction of lytic viral proteins leads to an increase in infectious virus or virus genomes. However, no lytic virus could be detected in lysates of whole lung tissue by plaque assay or by TCID_50_ assay, and the viral genomic load was found to be similar in all groups (Fig. 5a). We furthermore determined the expression of the viral genes ORF50 and ORF73 by RT-PCR in cells from bronchoalveolar lavage (BAL) and whole lung tissue as already described for the in vitro experiments. In whole lung tissue, the ORF50/ORF73 ratio increased slightly in mice after treatment with NP, indicating that ORF50 expression and thus lytic virus production might be induced (Fig. 5b), but it did not reach the values seen in acutely infected mice (Virus 6d: 3.2fold increase vs. Virus 29d). In BAL cells, the ORF50/ORF73 ratio showed a significant increase after exposure to CNP and a visible but not statistically significant increase after exposure to DWCNT, indicating that virus reactivation occurs in BAL cells (Fig. 5c). BAL cell analysis revealed the well-known acute inflammatory response to high surface area carbon nanoparticles and accordingly, the BAL cell composition changed most significantly by an increase in neutrophilic granulocytes from virtually zero for day 29 virus infected mice to 25.6 ± 10.7% for additionally CNP and 9.0 ± 1.5% for additionally DWCNT treated mice (Fig. 5d). In contrast, the lymphocyte percentage raised from 7.3 ± 2.3 to 8.9 ± 2.3% and 14.0 ± 2.2%, respectively. Taken together, our results suggest that NP exposure of latently infected mice apparently induced an abortive virus reactivation which led to an increased expression of lytic viral proteins but not to the generation of new infectious virus.

### Short-time treatment of latently infected mice with NP creates a transcriptome signature with parallels to acute virus infection

Next, we investigated if there is a match in the lung transcriptome between acutely infected mice and latently infected mice treated with NP. For that reason, total RNA from lung samples was isolated, processed and analyzed with the Illumina-MouseRef-8v2.0 Expression BeadChip as described in Materials and Methods. We scanned the data for genes that were both regulated in the acute infection situation and in latent infection plus NP compared to untreated mice and mice with latent virus alone. In the group “latent infection plus CNP”, we found an overlap between this group and the group of acutely infected mice of 208 significantly altered genes (116 upregulated and 92 downregulated) from which 34 genes were at least 1.5-fold changed compared to the control groups (30 upregulated, 4 downregulated; Fig. 5e). Some of the induced genes (such as Saa3, Timp1, Cxcl1, Slc26a4, Lcn2, Ch25h and Cd14) have recently been described to be also induced by instillation of a single high dose of 162 μg CNP in the lungs of mice [40]. 18 genes including the aforementioned showed upregulation in mice with CNP only in our experiments as well, even after exposure to the comparatively moderate dose of 50 μg (Additional file 1: Table S1). As differential expression of these genes is also detected during acute virus infection, they apparently are representatives of general pathways of inflammation and immune response that are both induced during acute infection and other inflammatory events. To test if the differentially expressed genes indeed represented specific cellular pathways, data were analyzed by the use of Ingenuity Pathway Analysis (IPA, https://www.ingenuity.com). As expected, functional pathways found to be differentially regulated in latently infected mice treated with CNP as well as in acutely infected mice involved immune functions such as “activation of leukocytes” or “invasion of cells”, but also pathways associated with cell proliferation (Fig. 5g). In the group “latent infection plus DWCNT”, the overlap with the group of acutely infected mice consisted of 369 significantly altered genes (156 upregulated, 213 downregulated) and expression of 49 of these genes was at least 1.5-fold changed compared to the control groups (43 upregulated; 6 downregulated; Fig. 5f). Similarly to the results observed in mice treated with CNP, 19 of these 49 genes were also regulated by DWCNT alone (Additional file 1: Table S1). Data analysis using IPA showed an increase in inflammatory pathways and a reduction in “import of D-glucose” (Fig. 5h).

Additionally, we analyzed the transcriptome data by an unbiased approach for differential regulation of genes after combination of latent virus and particle exposure compared to all control groups, and heatmaps showing these genes were generated (Additional file 1: Fig. S5). In the group treated with virus and CNP, 257 differentially regulated genes were detected (110 upregulated, 147 downregulated) and 38 of these genes showed at least 1.5-fold changed expression values compared to all control groups (35 upregulated, 3 downregulated; Additional file 1: Fig. S5a). Data analysis using IPA revealed differential regulation of pathways associated with migration, activation and proliferation of cells (Additional file 1: Fig. S5c). In the group treated with virus and DWCNT, 881 differentially regulated genes were found (288 upregulated, 593 downregulated), and 197 of these genes were at least 1.5-fold changed compared to controls (92 upregulated, 105 downregulated; Additional file 1: Fig. S5b). Data analysis with IPA depicted regulation of pathways involved in proliferation and organization of cells, but also tumor-associated pathways (Additional file 1: Fig. S5d). In contrast, the IPA pathways assigned to only CNP or DWCNT (24 h) treated mice depicted mainly the inflammatory pathways: inflammatory response, cell movement, leukocyte migration and recruitment of leukocytes (data not shown). The results obtained with the Illumina expression BeadChip were validated by quantitative real-time PCR for 6 selected genes (Additional file 1: Fig. S6).

Our transcriptome data indicates that exposure of latently infected lungs to NP creates a unique expression profile which cannot be observed in untreated controls or in latently infected mice without a second hit and only to some extent in mice that were exposed to NP only. The observed transcriptome signature shows similarities to the one seen in acute virus infection and is characterized by the stimulation of inflammatory processes and the induction of an immune response.

### Short-time treatment of latently infected mice creates a metabolite pattern in lung tissue with high similarity to the metabolite composition in acute virus infection

**Fig. 6:**
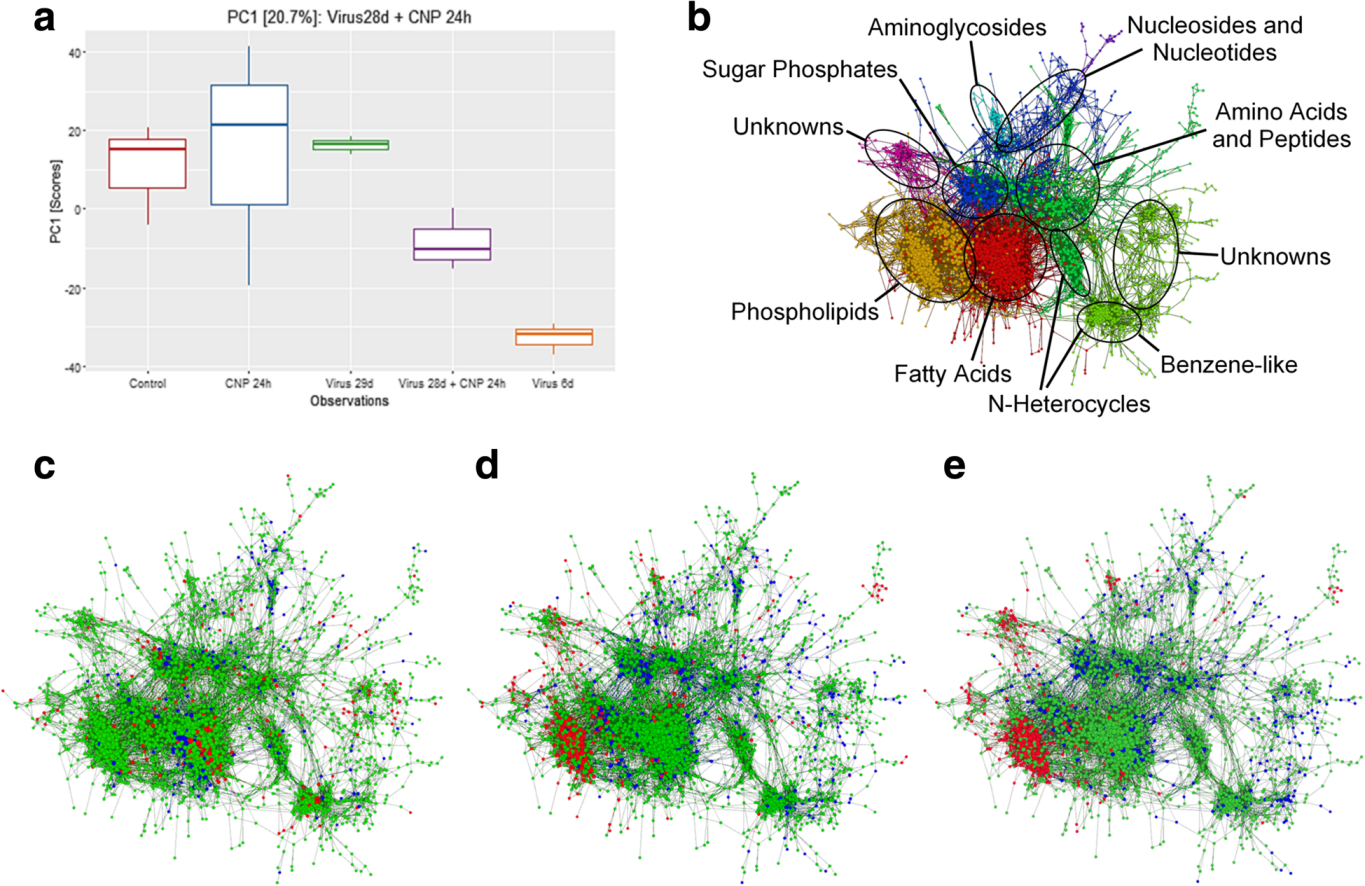
Exposure of latently infected mice to NP produces a metabolite composition in the lung resembling the one seen in acute virus infection: PCA was used to compare chemical similarities in the examined groups, and significant observations on PC-scores are shown in panel **a**. Mass difference networks were created to visualize the chemical similarity of the combined virus plus CNP treatment and the control groups. Figure 6 **b** gives an overview of the compound classes shown in panels **c**-**e**. The mass difference networks of the two control groups “virus alone” and “CNP alone” appeared exactly the same but proved to be entirely contraindicative once performed separately with compounds that were upregulated in one group being downregulated in the other group **c**. Green color stands for unchanged compounds. Blue color represents metabolites that are upregulated in “virus 29d” and downregulated in “CNP 24 h”. Red color depicts metabolites that are upregulated in “CNP 24 h” and downregulated in “virus 29d”. The pattern observed in “virus 28d + CNP 24 h” **d** showed upregulation of specifically one compound group. This profoundly resembles the pattern seen in acute virus infection **e**. In panels d and e, blue color stands for downregulated metabolites while red color depicts upregulated ones. The most pronounced changes in the metabolic profile were seen in the group of phospholipids. All data are obtained from 3 mice per group

In order to examine chemical similarities between the experimental groups, comparisons were performed on subsets of the mass spectrometric data using principal component analysis (PCA; see also Additional file 2). Loadings into the first two PCs highlighted a strong impact of glycerophospholipids into the acute viral infection metabotype. The first PC loadings of the second-hit-scenario “Virus 28d + CNP 24 h” (covering 20% of covariance) correlated with the acute virus infection metabotype (Fig. 6a; *p* = 2.37 × 10^−4^). A more detailed view of the compound class pattern shown in Fig. 6a as defined by the corresponding PC-loadings is shown in Additional file 1: Fig. S7. Querying the HMDB 3.5 database [41] for all 2697 annotated molecular formulas resulted in 642 hits (covering 23.8% of all annotated molecular formulas) and revealed that the acute virus infection phenotype is majorly characterized by the overrepresentation of glycerophospholipids and organic phosphoric acids as well as an underrepresentation of monosaccharides, carboxylic acids, peptides, steroids, amino acids and prenol lipids.

In order to illustrate the involvement of their corresponding compound classes with the found multivariate associations, a mass difference network (MDiN) was created (Fig. 6c-e) in which nodes are the metabolite candidates and edges are formula differences (potential biochemical reactions). Fig. 6b visualizes the compound classes shown in the metabolite networks. As implied by the similarity of their loadings with the acute viral infection metabotype, the combined treatment with latent virus plus CNP showed high similarity to the pattern seen in acute virus infection, characterized by an upregulation of phospholipids, but not to the control groups (Fig. 6d and e and Additional file 1: Fig. S7).

More detailed information on the involved building blocks could be mined using mass difference enrichment analysis (MDEA; as described by Moritz et al. [42]). In MDEA, MDiN-edges are interpreted as biochemical reactions or building blocks, whose association to nodes of interest can be investigated by the discrete Fisher’s exact test. MDEA results implied the induction of various fatty acid pathways as the major building blocks of acute viral infection and markers were C18-C22 saturated and poly-unsaturated fatty acids (Z-Scores and *p*-values ranged from 5.87 to 9.94 and 0 to 8.68 × 10^−8^, respectively). Confirming the data shown in the MDiN, one major upregulated compound class was found to be glycerophospholipids (see also Additional file 1: Fig. S7). Furthermore, arachidonic acid and eicosatrienoic acid molecular formula were determined significantly frequent, indicating a non-random usage of these substances and similar tendencies could also be found for other unsaturated fatty acids such as linoleic acid. On the other hand, down-regulated, or extensively consumed, metabolites were found to be composed of typical metabolites of glycolysis pathways. DWCNT exposure of latently infected mice caused a similar response to a certain extent, which was also accompanied by upregulation of phospholipids (Additional file 1: Fig. S8), but the response was not significant. This might be due to the strong hydrophobic nature of DWCNT which might adsorb compound groups such as phospholipids and therefore detract them from analysis. Taken together, the profound match of the metabolite pattern observed between acutely infected mice and latently infected mice treated with CNP suggests that – similar to our aforementioned observations – short-time treatment of latently infected mice with NP induces a boost of lytic virus replication and restores features of an acute virus infection in these mice.

### Latently infected B cells and macrophages are differentially affected by TiO_2_ NP and diesel exhaust particles (DEP)

To investigate if other types of commonly investigated low-toxicity low-solubility particles show a similar effect as described for CNP and DWCNT, we exposed latently infected cell lines to aqueous dispersions of commercial titanium dioxide NP (TiO_2_ NP, P25) or to diesel exhaust particles (DEP, SRM 2975) (Z-Average and PdI see Additional file 1: Fig. S1). The cells were treated with TiO_2_ NP or DEP for 72 h and virus titers in the supernatant and the ratio of ORF50/ORF73 expression were determined. Differences between the cell lines concerning the impact of the different types of NP were detected. Significant effects on virus reactivation were, however, only observed for DEP and the expression of the lytic switch protein ORF50 in S11 B lymphocytes (Additional file 1: Fig. S9a and c). Under these conditions, TiO_2_ NPs induced a similar pattern but did not reach statistical significance (*p* > 0.05). For ANA-1 macrophages, neither NP caused significant virus reactivation, i.e. increased the production of lytic virus and the expression of the viral gene ORF50 (Additional file 1: Fig. S9b and d). These comparisons suggest that – depending on the cell type – different nanomaterials might have different potencies to affect the maintenance and control of viral latency.

### Carbon NP reactivate EBV from latently infected human cells

**Fig. 7:**
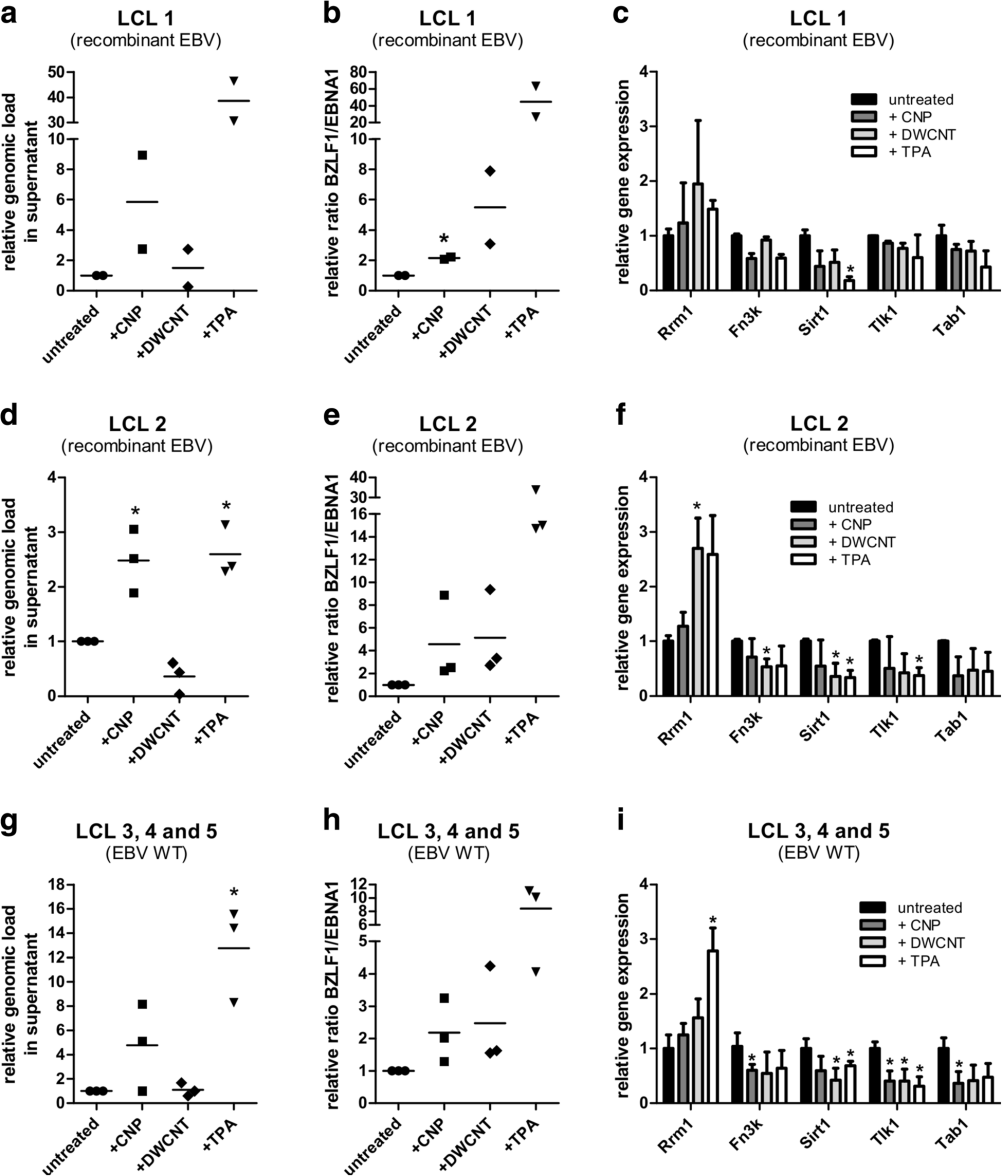
Exposure of persistently infected human cells to carbon NP induces reactivation of EBV: two lymphoblastoid cell lines (LCL) infected with recombinant EBV (panels **a**–**c** and **d**–**f**) and three LCL infected with EBV WT (panels **g**–**i**) were incubated with 50 μg/ml CNP or DWCNT for 72 h. Virus genomes in the supernatant were determined by qPCR for the viral gene EBNA1 after 72 h (panels **a**, **d** and **g**; individual values and means). Expression of the viral genes BZLF1 (specific for the lytic phase) and EBNA1 (expressed in all phases of EBV life cycle) – shown as the ratio BZLF1/EBNA1 (panels **b**, **e** and **h**; individual values and means) – and expression of genes that have been shown to be associated with reactivation of latent virus (panels **c**, **f** and **i**; means + SD) was analyzed by RT-PCR 72 h after NP exposure. The values in untreated cells were set as “1” and the values for cells after NP treatment were calculated relative to the control. The symbols shown in panels **a**–**c** depict two, and in panels **d**-**e** three independent experiments. In panels **g**-**h**, the symbols reflect three different cell lines tested in a single experiment. Asterisks indicate a statistically significant difference to the untreated control (*: *P* < 0.05)

We next analyzed whether reactivation of latent virus by exposure to NP also occurs in human cells latently infected with the human gammaherpesvirus EBV. As it is a well-known phenomenon in EBV-biology that different EBV-infected cell lines can vary highly in their response to stimuli [43, 44], we tested five different lymphoblastoid cell lines (LCL) that were either infected with recombinant EBV or EBV wildtype. The cells were incubated with CNP, DWCNT or TPA (used as a positive control) for 72 h, and viral genomes in the supernatant were quantified by qPCR. In addition, expression of the viral genes BZLF1 (= Zta), which reactivates the EBV lytic cycle [45] and EBNA1 (episome maintenance protein), which is particularly important in viral latency but is expressed throughout all phases of the viral life cycle [46], was determined by RT-PCR. Relative expression of BZLF1 as a marker for the induction of lytic virus production increased both after exposure to CNP and to DWCNT (Fig. 7b, e and h). Although the strength of the effect varied from cell line to cell line, the BZLF1/EBNA1 ratio was always increased after NP treatment. Consistent with the BZLF1/EBNA1 ratio, increased amounts of viral genomes in the supernatant of cells treated with CNP were detected by qPCR, confirming that new virus particles are produced (Fig. 7a, d and g). Surprisingly, no increase in viral genomes could be observed after treatment with DWCNT. This seems to contradict the results found for the BZLF1/EBNA1 ratio, but we assume that it might be a false-negative result as the strongly hydrophobic DWCNT might capture the DNA during the isolation procedure and thereby exclude it from the downstream analysis. This is in line with already published observations demonstrating the ability of DNA to bind to carbon nanotubes through pi-stacking and the strong hypochromic interactions of DNA with carbon nanotubes in aqueous media [47, 48]. We tried additional DNA isolation methods but so far, we were not able to overcome this problem. We furthermore tested the expression of the five previously described cellular genes with potential roles in virus reactivation (Rrm1, Fn3k, Sirt1, Tlk1 and Tab1). All genes showed the expected down- or upregulation (Fig. 7c, f and i), but to a varying extent and with some variation between the experiments. Therefore, statistically significant differences could only be found for some genes and conditions. Nevertheless, our results clearly indicate that the effect of carbon NP is not limited to murine cells or tissues but that exposure to NP also reactivates EBV from latently infected human cells.

## Discussion

Since both inhalation of environmental NP and persistent herpesvirus-infection have been implicated to contribute to the development of chronic lung disease, we hypothesized that the combination of both might lead to a different outcome than each factor alone. Given that virtually every human being is persistently infected with herpesviruses, NP exposure of an already infected individual can be easily envisaged as a practically relevant scenario. At present, no information about the health relevance of a suchlike liaison of NP and persistent virus infection is available.

There are several publications providing evidence that the presence of carbon-based NP has an influence on virus infection in mice or in cells. For example, it has been demonstrated that exposure of cells to single-walled carbon nanotubes increases the susceptibility of these cells to infection with influenza H1N1 [49]. Additionally, it has been shown that preexposure of mice to carbon black prior to infection with respiratory syncytial virus (RSV) induces an inflammatory milieu that promotes disease exacerbation [50]. Furthermore, treatment of RSV-infected mice with ultrafine carbon black enhances the expression of various chemokines that are associated with virus infection, and leads to an enhanced RSV-induced airway hyperresponsiveness to methacholine [51]. In this paper, we show that NP exposure of persistently herpesvirus-infected cells in vitro reactivates latent virus. Interestingly, we observed differences depending on the cell type (B cells, macrophages) and on the type of NPs (CNP, DWCNT, TiO_2_, or DEP). For example, the effect of DWCNT was more pronounced than the one by CNP in most of our experiments. This does not seem to be a consequence of the higher surface area but might be due to the fact that DWCNT, apart from inducing oxidative stress and activating acute inflammatory responses, affect a number of additional cytotoxicity pathways [52]. The triggers of herpesvirus reactivation and the underlying molecular mechanisms are – taken as a whole – only incompletely understood [53, 54], and along the same line, we so far can neither depict by which mechanisms/pathways NP might induce reactivation of latent virus nor which are the target cells for this interaction in vivo. In our in vivo experiments we could show that NP exposure of persistently infected mice leads to the expression of lytic viral proteins and restores a signature observed during acute virus infection. Although we observed the induction of lytic viral proteins after NP treatment of latently infected mice, no increase in infectious virus or virus genomes could be detected. This might indicate that only small amounts of new infectious virus were produced which were below the detection limits of the assays used. Another possibility is that NP treatment induces an abortive reactivation which leads to re-expression of lytic virus proteins – serving as potential targets for the immune system – but does not lead to the completion of the replication cycle and the production of new infectious virus. Such a repetitive appearance of viral proteins induced by NP-exposure might nevertheless provoke the infiltration of immune cells and finally cause a chronic aberrant inflammatory response even in the absence of completely assembled infectious virus. For example, it has been shown that the CD8^+^ T cell response against MHV-68 antigens can mediate inflammation and altered cellular recruitment to the lung, finally resulting in immunopathology and fibrosis [55]. The increased appearance of glycerophospholipids which was found by metabolome analysis in the lungs of acutely infected mice as well as in the lungs of latently infected mice treated with NP is another indicator for the presence of lytic virus replication – irrespective of successful completion of the replication cycle or not. Noteworthy, the phospholipid pattern was observed in association with the detection of lytic MHV-68 proteins only, but not with the acute inflammatory response caused by NP exposure alone. As shown by Sutter et al., who investigated the role of phospholipids in infection with Herpes simplex virus, virus infection triggers the production of phospholipids to maintain cellular membrane integrity and to deliver membrane components for envelopment of virus capsids and formation of transport vacuoles during virus production [56]. Other substances detected by metabolome analysis such as arachidonic acid, eicosatrienoic acid, and linoleic acid imply upregulation of mediator molecules of immunomodulatory and of oxidative stress related pathways after exposure of latently infected mice to CNP. Particularly arachidonic and eicosatrienoic acid have been described to have an impact on primary infection with herpesviruses as well as on virus reactivation [57].

## Conclusions

In this paper, we show that exposure of latently infected cells or tissues to NP leads to reactivation of latent virus accompanied by an increase in viral proteins and metabolome- and transcriptome-signatures that can also be found in acute virus infection. Concerning the health relevance for humans it should be considered that application of a single dose of NP, as in our experiments, does only partially reflect the occupational settings where the applied amount might be gradually accumulated over one working week. Nevertheless, repetitive appearance of even a small amount of viral proteins, induced by exposure to NP, might be sufficient to trigger a chronic aberrant immune response and consequently lead to tissue damage. The question whether the combined exposure to NP and virus de facto causes disease aggravation needs to be further investigated and will be a focus of subsequent studies.

## Methods

### Nanoparticles

Four different types of NP were used in this study (see also Table 1): carbonaceous spherical NP (CNP; Printex90, Degussa, Frankfurt, Germany), double-walled carbon nanotubes (DWCNT; Nanocyl, Auvelais, Belgium), TiO_2_ NP (P25, Degussa, Frankfurt, Germany), and diesel exhaust particles (DEP; SRM 2975, National Institute of Standards and Technology, Gaithersburg, MD, USA). CNP, TiO_2_ and DEP were suspended in pyrogen-free water using a previously described method [5]. For in vitro experiments, DWCNT were suspended in medium, incubated in an ultrasonic bath and subsequently sonicated on ice for 1 min prior to use, using a Bioruptor (Diagenode, Liege, Belgium). For in vivo experiments, DWCNT were dispersed in pyrogen-free, distilled water supplemented with 1% Pluronic F-127 (Sigma-Aldrich, Germany) – an FDA approved surfactant to facilitate dispersion quality - as described earlier [58]. The average size (Z-Ave) and size distribution (represented by the polydispersity index = PdI) of nanoparticles dispersed in medium and in water was determined by photon correlation spectroscopy using a Dynamic Laser Scatter (DLS) Zetasizer Nano ZS. (Malvern Instruments Ltd., Malvern, UK) as described in the literature [59]. The dispersion quality is shown in Additional file 1: Fig. S1. The absence of endotoxin from the particle preparations was approved by LIMULUS assay (<0.05 EU/μg CNP or DWCNT), and even more relevant by in vitro studies using an LPS-sensitive alveolar macrophage cell line which showed no pro-inflammatory activation (e.g. TNFα gene and protein expression) for concentrations up to 150 ug/ml CNP or DWCNT.

**Table 1:** Nanoparticles used in this study

Type	Name	Acronym	Size ^a^ [nm]	BET [m^2^/g]	Source
Carbon black	Printex90	CNP	14	272	Evonik Degussa GmbH
Carbon nanotubes	DWCNT	DWCNT	10 × 1000	660	Nanocyl
TiO_2_	P25	TiO_2_	21	50	Evonik Degussa GmbH
Diesel exhaust particles	SRM 2975	DEP	32	91	NIST

Overview of features of the nanoparticles used in this study (all data according to manufacturer’s information; Size ^a^: average primary particle size)

### Cell lines

BHK-21 cells (ATCC: CCL-10) were grown in Glasgow-MEM (PAN Biotech, Aidenbach, Germany) supplemented with 5% fetal calf serum (FCS; PAN Biotech, Aidenbach, Germany), 5% tryptose phosphate broth, 2 mM L-glutamine, 100 U/ml Penicillin and 100 μg/ml Streptomycin. NIH 3T3 cells (ATCC: CRL-1658) were grown in DMEM High Glucose (Gibco, Darmstadt, Germany) supplemented with 5% FCS, 2 mM L-Glutamine, 100 U/ml Penicillin and 100 μg/ml Streptomycin. The persistently with MHV-68 infected B cell line S11 [30], the two macrophage cell lines ANA-1 [60, 61] and MH-S (ATCC: CRL-2019), and human LCL cell lines (kindly provided by Bettina Kempkes and Josef Mautner, Helmholtz Zentrum Muenchen, Munich, Germany) were cultivated in RPMI (Gibco, Darmstadt, Germany) supplemented with 15% fetal calf serum (FCS; PAN Biotech, Aidenbach, Germany), 2 mM L-glutamine, 1% non-essential amino acids (Gibco, Darmstadt, Germany), 100 U/ml Penicillin and 100 μg/ml Streptomycin. For S11 and MH-S cells, 50 μM 2-Mercaptoethanol (Bioconcept, Allschwil, Switzerland) was added to the medium.

### Determination of cell viability by WST assay

Cell viability after exposure to NP was measured by WST assay, which determines the activity of mitochondrial succinate dehydrogenase in cells, according to the manufacturer’s instructions (Roche Diagnostics, Mannheim, Germany). Briefly, cells were plated and incubated with the indicated concentrations of NP for 72 h. WST reagent was added and incubated with the cells for 2 h at 37 °C. The plates were centrifuged to remove the bulk of NP agglomerates and supernatants were transferred to a new plate prior to analysis. Enzymatic conversion of WST reagent was determined using an ELISA-reader at 430 nm with 630 nm as reference.

### Analysis of lytic virus growth in vitro after NP treatment

To test lytic growth of MHV-68 in vitro after NP treatment, MH-S cells or LA-4 cells were infected with a MOI of 1 for 2 h. After removing the inoculum (=0 h), cells were washed two times with PBS and then treated for 2 h with 50 μg/ml NP (NP preparation see section “Nanoparticles”). After the incubation period, the inoculum was removed, the cells were washed two times with PBS and then incubated with fresh medium at 37 °C and 5% CO_2_ until the supernatants or the supernatants together with the cells were harvested at different time points after infection. Virus titers were determined by plaque assay on BHK-21 cells.

### In vitro assay for measurement of low level virus replication in infected macrophages

To measure low-level virus replication in infected macrophages, a modification of a previously described method for a limiting dilution in vitro reactivation assay was used [28]. Briefly, MH-S cells (alveolar macrophage cell line) were plated in a 6-well plate and infected with MHV-68 o.N. at an MOI of 0.01. The inoculum was removed and each well was washed two times in PBS. The cells were then either left untreated or incubated with 50 μg/ml CNP or DWCNT (NP preparation see section “Nanoparticles”). After 2 h, the medium containing the NP was removed and each well was washed two times with PBS. The cells were incubated for 1 min with an acidic citrate buffer (pH = 3.0) to remove remaining lytic virus from the inoculum and washed three times with medium. Serial threefold dilutions of infected MH-S cells were plated on monolayers of 7 × 10^3^ low-passage NIH 3T3 cells per well in 96-well tissue culture plates. Twenty-four wells were plated per dilution (starting with 1 × 10^3^ MH-S cells). NIH 3T3 cells were screened microscopically for a viral cytopathic effect for up to 2 weeks. To differentiate between freshly produced virus and residual lytic virus from the inoculum, serial threefold dilutions of MH-S cells were plated before or after mechanical disruption of viable cells (by two freeze-thaw cycles). Frequencies of cells supporting lytic virus replication were calculated on the basis of the Poisson distribution by determining the cell number at which 63.2% of the wells scored positive for CPE. To compensate for variations in the infection efficiency, the viral genomic load was determined as described below and taken account of when calculating the frequency of cells producing lytic virus.

### Generation of a persistently infected macrophage cell line

To generate a persistently infected macrophage cell line, we constructed a recombinant MHV-68 containing a hygromycin-resistance cassette by a two-step mutagenesis procedure using the BAC-technology [62, 63]. To this end, a 2.4 kb expression cassette containing the coding sequence of hygromycin phosphotransferase driven by the SV40 early-enhancer promoter, was excised from vector pRTS-1 [64] (kindly provided by Bettina Kempkes, Helmholtz Zentrum Muenchen, Munich, Germany) and cloned blunt end into the PmlI site (nucleotide position 46.347 of the MHV-68 genome) of the plasmid pST76K-SR already containing a 4.0 kb SphI-SacI fragment of MHV-68 (nucleotide positions 44.301 to 48.346). As a result, the hygromycin phosphotransferase expression cassette is flanked on both sides by homologous sequences as needed for homologous recombination during the two-step mutagenesis procedure. BHK-21 cells were transfected with 1.5 μg of BAC MHV-68-Hygro DNA using X-treme GENE HP DNA Transfection Reagent (Roche, Mannheim, Germany) to reconstitute recombinant MHV-68-Hygro. A virus stock was generated and the virus titer was determined by plaque assay on BHK-21 cells. To establish a permanently infected cell line, the bone marrow derived macrophage cell line ANA-1 was infected with MHV-68-Hygro at an MOI of 1. Hygromycin B (Sigma-Aldrich, Seelze, Germany) at a final concentration of 500 μg/ml was added 24 h after infection and persistently infected cells were expanded under permanent selection. As the BAC-sequence in the virus genome contains the GFP-gene, the cells could be monitored under the fluorescence microscope and more than 90% of the cells proved to be GFP positive.

### Treatment of persistently infected cell lines with NP

To analyze the effect of NP exposure on persistently infected murine cells, the B cell line S11 and the macrophage cell line ANA-1/MHV-68 were used. To investigate the effect of NP on persistently infected human cells, human lymphoblastoid cell lines (LCL) were used. NP were suspended as described above and added to the cells at a concentration of 5 μg/ml or 50 μg/ml. After 72 h, supernatants were harvested for analysis of virus titer by Plaque assay (murine cells) or qPCR (human cells), and cells were harvested for RNA isolation and subsequent RT-PCR.

### Measurement of viral genomic load by quantitative real time PCR

DNA was isolated from lung tissue samples that were homogenized by using the FASTPREP^®^-24 instrument (MP Biomedicals, Heidelberg, Germany), from cell culture supernatants, or from infected cell lines with the QIAmp DNA Mini Kit (Qiagen, Hilden, Germany). The viral genomic load in infected murine cells or in murine lung tissue was determined by quantitative real-time PCR using the ABI 7300 Real Time PCR System (Applied Biosystems, Foster City, CA) as described previously [65]. The amount of viral genomes in cell culture supernatants from LCL was analyzed by real time quantitative PCR for the viral gene EBNA1 using the Taqman SYBR green PCR master mix (Applied Biosystems, Foster City, CA).

### RT-PCR

mRNA was isolated from cells or tissues using the RNeasy MiniKit (Qiagen, Hilden, Germany) including DNase digestion of remaining genomic DNA. RNA was reverse-transcribed using the High Capacity cDNA Reverse Transcription Kit (Applied Biosystems, Foster City, CA). The cDNA was analyzed for the expression of selected genes by real time quantitative PCR using the Taqman SYBR green PCR master mix (Applied Biosystems, Foster City, CA). The used primer sets are depicted in Table 2. The fold change in expression of each target mRNA relative to beta-Actin (Actb, murine cells) or HPRT (human cells) was calculated based on the threshold cycle (Ct) as 2^-ΔCt^, where ΔCt = Ct_specific gene_ – Ct_beta-Actin_. The mean value for untreated controls was set as 1, and all values were calculated relative to the control.

**Table 2:** Primer sets used in this study

Gene name	Forward primer (5’–3’)	Reverse primer (5’–3’)
Acta2	GTCCCAGACATCAGGGAGTAA	TCGGATACTTCAGCGTCAGGA
Actb	TCCATCATGAAGTGTGACGT	GAGCAATGATCTTGATCTTCAT
Bex2	TCCAAAGTGGAACAAGGCGT	GCACGTAGTAGTCTCCAGCTTC
Fn3k	TGGCCCCGTGTTTGTCAAG	CTGGCAAGTCAATCACCTTCAT
Ltbp4	CTGGGTGTCGCTATTGGTG	GTTGTGACAGATCAAGGGACAT
Tab1	CTTTCGCAACTGGGTTTAGACG	CGCCATGAATTTCCGGCTC
MHV-68 ORF50	CCCACGGTTCGCTATACAGTAAAGAC	ATTGTGTAGAGGGTCCAGGTTAATGATTTC
MHV-68 ORF73	TGGTGGAGGAGGGGCTGGTC	ACCGACTACACGCAACACAACC
Pvalb	ATCAAGAAGGCGATAGGAGCC	GGCCAGAAGCGTCTTTGTT
Rrm1	CTGGCAGCAAGGATAGCCG	CCGTTGTGCGGATTTATGTAGT
Sirt1	ATGACGCTGTGGCAGATTGTT	CCGCAAGGCGAGCATAGAT
Spp1	AGCAAGAAACTCTTCCAAGCAA	GTGAGATTCGTCAGATTCATCCG
Sprr1a	TTGTGCCCCCAAAACCAAG	GGCTCTGGTGCCTTAGGTTG
Tlk1	AGTCAGGGAAAAAGTATCGGGG	TTCTGCGGAGAACGGATTGC
EBV EBNA1	CCAAGAAGGTGGCCCAGA	CCTGCCTCCATCACCCTG
EBV BZLF1	TTCCACAGCCTGCACCAGTG	GGCAGCAGCCACCTCACGGT
humanFn3k	CATCCCGCAGGTGAATGAGTG	GGGGACAATCTCTAGGCCAC
humanHPRT	GCAGACTTTGCTTTCCTTGGTCAG	GTCTGGCTTATATCCAACACTTCGTG
humanTab1	TGAGGAACTTTGGCTACCCG	GTCGGGCTTTGGTTGGTGA
humanRrm1	CATGTGATCAAGCGAGATGGC	GTGACCCCACTGTACAAGCC
humanSirt1	TGGGTACCGAGATAACCTTCT	TGTTCGAGGATCTGTGCCAA
humanTlk1	CCATCTTGGTCCCAGCTCTCC	CGTTATTTGTTGAGGCTTTAGCTCC

### In vivo experiments

C57BL/6 mice were purchased from Charles River Laboratories (Sulzfeld, Germany) and housed in individually ventilated cages (IVC) during the MHV-68 infection period. Mice were anesthetized with ketamine and xylazine and infected i.n. with 5 × 10^4^ PFU MHV-68. After 28 days, mice were either left untreated or instilled with 50 μg of spherical (CNP) or double-walled fibre-shaped carbon nanoparticles (DWCNT) per mouse as described earlier [5, 58] (NP preparation see section “Nanoparticles”). Lung tissue was harvested after 24 h for transcriptome and metabolome analysis as well as for determination of viral genomic load and for histology. Bronchoalveolar lavage (BAL) cells for RNA isolation and analysis of viral gene expression were collected by cannulating the trachea and rinsing the lung six times with PBS as described earlier [26]. All animal experiments were in compliance with protocols approved by the local Animal Care and Use Committee (District Government of Upper Bavaria; permit numbers 124/08 and 67/2015).

### Metabolomics

For the metabolomics analysis, lung tissue samples (three mice per group) were processed by using the FASTPREP^®^-24 instrument (MP Biomedicals, Heidelberg, Germany). 50 mg of lung tissue were homogenized in 1 ml of ice-cold methanol (LC/MS grade, Sigma-Aldrich, Steinheim, Germany). The homogenates were centrifuged at maximum speed in an Eppendorf centrifuge to remove debris. The supernatants were stored at −80 °C and diluted 1:50 in methanol prior to metabolome analysis. Samples were injected in a randomized order at a flowrate of 120μLh^−1^ using a Gilson autosampler system (Gilson, Inc., Meddleton, WI, USA). Electrospray ionization (ESI) was performed using an APOLLO II ion source (Bruker Daltonics GmbH, Bremen, Germany) in negative ionization mode with capillary voltage and spray shield voltage being set to −3000 V and 500 V, respectively. Drying gas flow rate and temperature were set to 4Lmin^−1^ and 200 °C. The nebulizer pressure was set to 1.1 bar. Ultra-high resolution and accuracy mass spectrometra were recorded using a Bruker (Bruker Daltonics GmbH, Bremen, Germany) solariX Fourier Transform Ion Cyclotron Resonance Mass Spectrometer (FT-ICR-MS) equipped with a 12 T superconducting magnet. External calibration of the mass spectrometer is performed daily using a 1 mgL^−1^ arginine/methanol solution. Linear calibration on at least 4 arginine clusters is accepted once the standard deviation of m/z error was minor 100 ppb. Mass spectra were recorded over a mass range of 129 m/z to 1000 m/z. The time domain was set to 4 M words (MW) and the resolution at m/z = 400 was R = 430,000.

Raw mass spectra were pre-processed using Data Analysis Version 4.1 (Bruker Daltonics GmbH, Bremen, Germany). All mass spectra were uploaded and peak picking was performed using a signal to noise cutoff of S/N = 4. Linear calibration was performed using an in-house generated list of 390 metabolite m/z values that are frequently occurring across species and bio-fluids. The central error-m/z distribution was visualized within the Data Analysis calibration functionality and centered on zero ppm given a linear calibration function. The overall error standard deviation was found to be < 100 ppb. All calibrated mass spectra were exported as ASCI file and aligned with the in-house written software ‘Matrix Generator’ given an error window of 1 ppm.

M/z signals which were not once detected within a full triplicate were omitted. M/z peaks were subjected to combinatorial formula assignment at ±0.5 and given elemental counts of C_1-100_O_0-70_N_0-20_S_0-3_P_0–3_ using an in-house written software. Masses were filtered for the Senior rules and for diverse approximations of elemental relationships. Formulae were then run through a strict isotopic pattern matching algorithm assuming infinite resolution and given a noise level that was set to be the minimum of all maximal m/z intensities across all samples. Validated formulae were used as starting points for mass difference network-based formula assignment to low abundance peaks (> > 95% of a dataset) [66]. The initial dataset of >200.000 m/z values was reduced to 6890 m/z values by removal of non-triplicate features and reduced to 2697 sum formula annotations.

Data normalization was performed as follows: The inter quartile ranges (IQRs) of all non-zero entries per sample were calculated. The sample-wise Euclideans of all m/z feature intensities that were elements of their corresponding IQR were calculated and used for normalization.

Data was separated into two data sets which were composed of the second hit experiments (Virus 28d + CNP 24 h or Virus 28d + DWCNT 24 h), their corresponding time matched controls and a mouse-group with acute virus infection (Virus 6d).

Both datasets were scaled by Z-transformation and subjected to principal component analysis (PCA) using the Perseus software, version 1.5.1.6. The scores of the first three components of each dataset were tested for significant differences between combined exposure, single exposures and untreated controls using Student’s *T* test in Microsoft Excel 2016. Mass difference networks were reconstructed and mass difference enrichment analyses were performed using Matlab R2011 (as described in detail by Moritz et al.[42]). The theoretical mass difference network was reconstructed using 490 reaction equivalent mass differences (REMDs), part of which were derived from the KEGG metabolic maps and part of which were a manually curated extension and correction of data published previously by Breitling et al. [67]. Given theoretical molecular masses derived from molecular formula assignment, REMDs, which here are interpreted as building blocks, were used as edges to connect all annotations (nodes). REMDs were then tested for significant associations to metabolic features (molecular formulas) of interest using Fisher’s exact test, which assumes a hypergeometric distribution. This technique is used to include features that could not be matched to e.g. the human metabolome database (HMDB [41]). Box-plots were generated using ggplot2 in RStudio Version 0.99.489. Networks were visualized using Gephi-0.8.2.

### Immunohistochemical staining for lytic virus proteins

Expression of MHV-68 lytic proteins in the lung of NP-treated or control animals was examined by standard immunohistochemical methods. Since all lungs were divided for different assays, collapsed lungs had to be used for histology. Lung tissue was embedded in paraffin and cut into 4 μm sections. Slides were incubated with 3% hydrogen peroxide to bleach tissues and produce a better contrast for the alkaline phosphatase staining procedure. Following epitope retrieval by heating the sections in citrate buffer (pH = 6.0), the sections were incubated with blocking buffer (Rodent Block M; Biocare Medical/Zytomed Systems, Berlin, Germany) and labeled with a polyclonal rabbit serum directed against lytic proteins of MHV-68 (described previously by Steer et al. [68]; 1:500 dilution). After washing, Rabbit-on-rodent-AP-polymer was added (Biocare Medical/Zytomed Systems, Berlin, Germany), and finally, the phosphatase reaction was visualized using the Vulcan Fast Red Chromogen Kit (fuchsin-red reaction product; Biocare Medical/Zytomed Systems, Berlin, Germany). All sections were counterstained with hematoxylin.

### Transcriptome analysis

Total RNA from lung tissue was isolated employing the RNeasy MiniKit (Qiagen, Hilden, Germany) including DNase digestion of remaining genomic DNA. The Agilent 2100 Bioanalyzer was used to assess RNA quality and only high quality RNA was used for microarray analysis. 300 ng of high quality total RNA were amplified using the Illumina TotalPrep RNA Amplification kit (Ambion, Life Technologies, Carlsbad, CA, USA). Amplified cRNA was hybridized to Mouse Ref-8 v2.0 Expression BeadChips (Illumina, San Diego, CA, USA). Staining and scanning were done according to the Illumina expression protocol. Data was processed using the GenomeStudioV2010.1 software (gene expression module version 1.6.0) in combination with the MouseRef-8_V2_0_R3_11278551_A.bgx annotation file. The background subtraction option was used and an offset to remove remaining negative expression values was introduced. Data normalization (quantile) was performed by utilizing the statistical programming environment R implemented in CARMAweb [69, 70]. Genewise testing for differential expression was done employing the limma *t*-test and Benjamini-Hochberg multiple testing correction (FDR < 10%). Heatmaps showing genes that were at least 1.5fold regulated in mice treated with latent virus and NP compared to untreated control mice were generated with CARMAweb. Pathway enrichment analyses were done with the Ingenuity pathway analysis software (IPA®, Qiagen, Redwood City, CA, USA, https://www.qiagen.com/ingenuity). For genes that were detected by more than one probe, only one representative value is shown. Array data has been submitted to the GEO database at NCBI (GSE79501).

### Statistical methods

Datasets were analyzed by Student’s *t*-test using the GraphPad Prism software, vs5 (GraphPad Software, Inc., San Diego, CA, USA). Results with a *p*-value < 0.05 were considered significant. Statistical analysis of transcriptome data was performed as described in the section “transcriptome analysis”.

## Additional files

Additional file 1: Figure S1. Average Size and size distribution of the used NP. **Figure S2.** Measurement of cell viability. **Figure S3.** Exposure to NP reactivates lytic virus in persistently infected cells in vitro in a dose dependent manner. **Figure S4.** Exposure to NP reactivates lytic virus in persistently infected cells independently of the particle aspect ratio. **Figure S5.** Short-time exposure of latently infected mice to NP differentially regulates gene expression in whole lung tissue cells independently of the particle aspect ratio. **Figure S6.** Confirmation of gene expression data by real-time quantitative PCR for selected genes. **Figure S7.** Exposure of latently infected mice to CNP leads to an increase in glycerophospholipids. **Figure S8.** Exposure of latently infected mice to DWCNT leads to an increase in glycerophospholipids. **Figure S9.** Exposure of persistently infected cells to TiO2 NP or DEP has differential effects on virus reactivation in vitro. **Table S1.** Gene expression values of selected genes (PDF 1767 kb)

Additional file 2: Supplementary Material (XLSX 7772 kb)

## Abbreviations

BAL: Bronchoalveolar lavage
CNP: Carbonaceous nanoparticles
CPE: Cytopathic effect
d: Day
DEP: Diesel exhaust particles
DWCNT: Double-walled carbon nanotubes
EBV: Epstein-Barr-virus
h: Hour
HCMV: Human cytomegalovirus
IPF: Idiopathic pulmonary fibrosis
KSHV: Kaposi’s sarcoma-associated herpesvirus
LCL: Lymphoblastoid cell line
LPS: Lipopolysaccharide
MDEA: Mass difference enrichment analysis
MDiN: Mass difference network
MHV-68: Murine gammaherpesvirus 68
NP: Nanoparticles
PCA: Principal component analysis
RT-PCR: Reverse transcription polymerase chain reaction
TCID50: Tissue culture infective dose (amount of a pathogenic agent that will produce pathological change in 50% of cell cultures inoculated)
TiO2: Titanium dioxide
TPA: 12-O-tetradecanoyl-phorbol-13-acetate
