# Supplementary material for: Nanoparticle exposure reactivates latent herpesvirus and restores a signature of acute infection
Source: Part Fibre Toxicol. 2017 Jan 10;14:2. doi: 10.1186/s12989-016-0181-1 (PMC5223553; doi:10.1186/s12989-016-0181-1)
Supplement: Supplementary file 1 — Average Size and size distribution of the used NP. Figure S2. Measurement of cell viability. Figure S3. Exposure to NP reactivates lytic virus in persistently infected cells in vitro in a dose dependent manner. Figure S4. Exposure to NP reactivates lytic virus in persistently infected cells independently of the particle aspect ratio. Figure S5. Short-time exposure of latently infected mice to NP differentially regulates gene expression in whole lung tissue cells independently of the particle aspect ratio. Figure S6. Confirmation of gene expression data by real-time quantitative PCR for selected genes. Figure S7. Exposure of latently infected mice to CNP leads to an increase in glycerophospholipids. Figure S8. Exposure of latently infected mice to DWCNT leads to an increase in glycerophospholipids. Figure S9. Exposure of persistently infected cells to TiO2 NP or DEP has differential effects on virus reactivation in vitro. Table S1. Gene expression values of selected genes (PDF 1767 kb) [file 12989_2016_181_MOESM1_ESM.pdf]

Suspended in H_2O — Suspended in cell culture medium

a) CNP

b) DWCNT

c) DEP

d) TiO_2

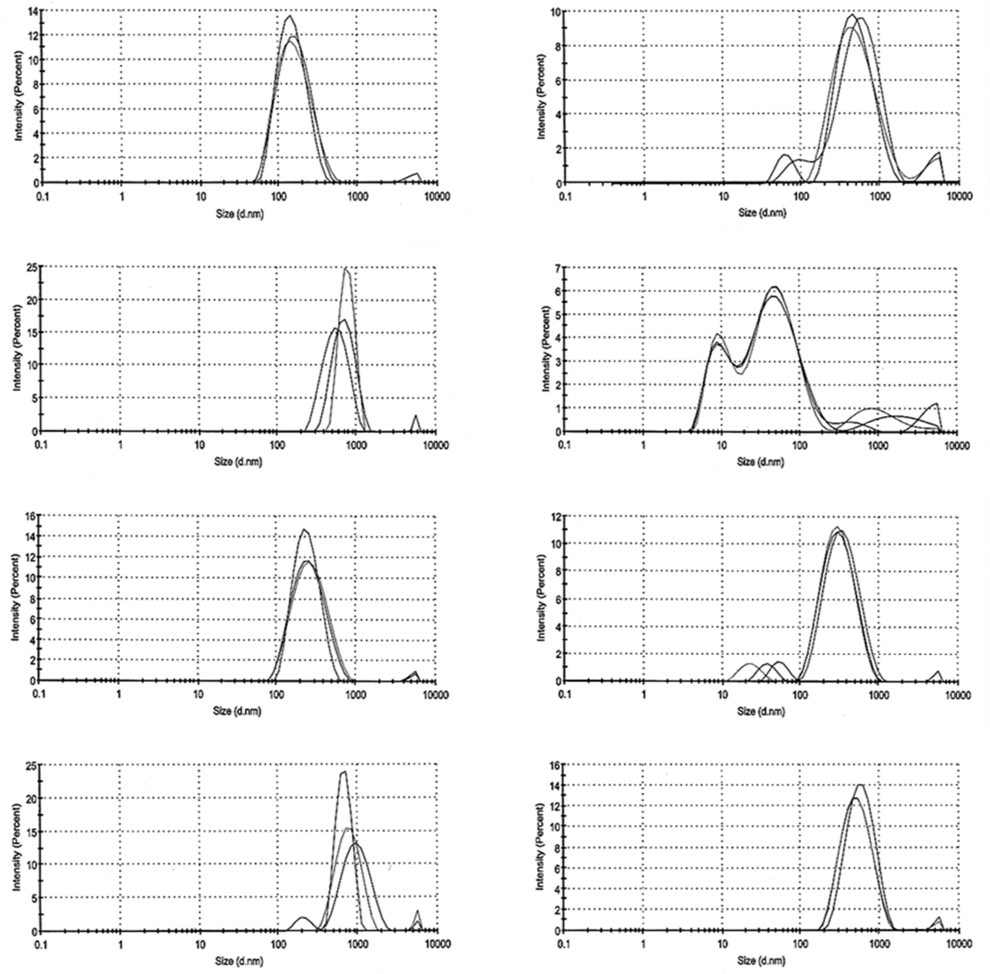

e)

particle type	suspended in…	Z-Ave [nm]	SD	PdI	SD
CNP	medium	337	4.66	0.52	0.02
CNP	H_2O	140	1.10	0.17	0.02
DWCNT	medium	26	1.00	0.57	0.09
DWCNT	H_2O	897	161.65	0.49	0.05
Diesel exhaust particles	medium	206	6.95	0.51	0.01
Diesel exhaust particles	H_2O	230	1.80	0.21	0.02
TiO_2	medium	427	22.36	0.37	0.04
TiO_2	H_2O	878	67.88	0.38	0.11

Figure S1. Average Size and size distribution of the used NP: The average size (Z-Ave) and size distribution (represented by the polydispersity index = PdI) of nanoparticles dispersed in medium and in water was determined by photon correlation spectroscopy using a Dynamic Laser Scatter (DLS) Zetasizer Nano ZS. The dispersion quality is shown in panels a – d. A summary of the obtained results can be found in the table in panel e.

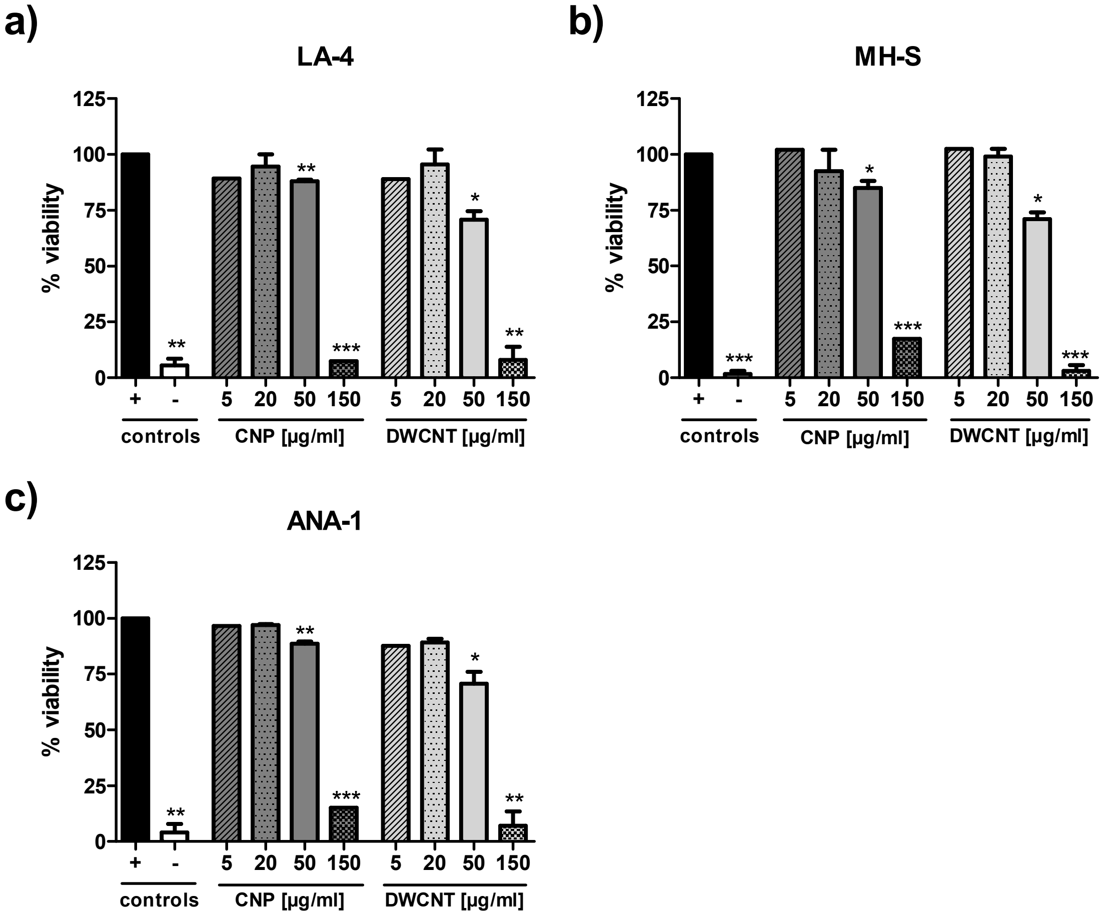

Figure S2. Measurement of cell viability: Cell viability after exposure to NP was measured in LA-4 cells (a), MH-S cells (b) and ANA-1 cells (c) by WST assay. Untreated cells ("+") and cells treated with 10% DMSO ("-") were used as controls. Data shown are the means + SEM from three experiments (controls, CNP 50µg/ml, DWCNT 50mg/ml, DWCNT 150µg/ml), two experiments (CNP 20µg/ml, CNP 150µg/ml, DWCNT 20µg/ml) or one single experiment (CNP 5µg/ml, DWCNT 5µg/ml) each measured in triplicates. Asterisks indicate a statistically significant difference to the untreated control (*: $P < 0.05$; **: $P < 0.01$; ***: $P < 0.001$).

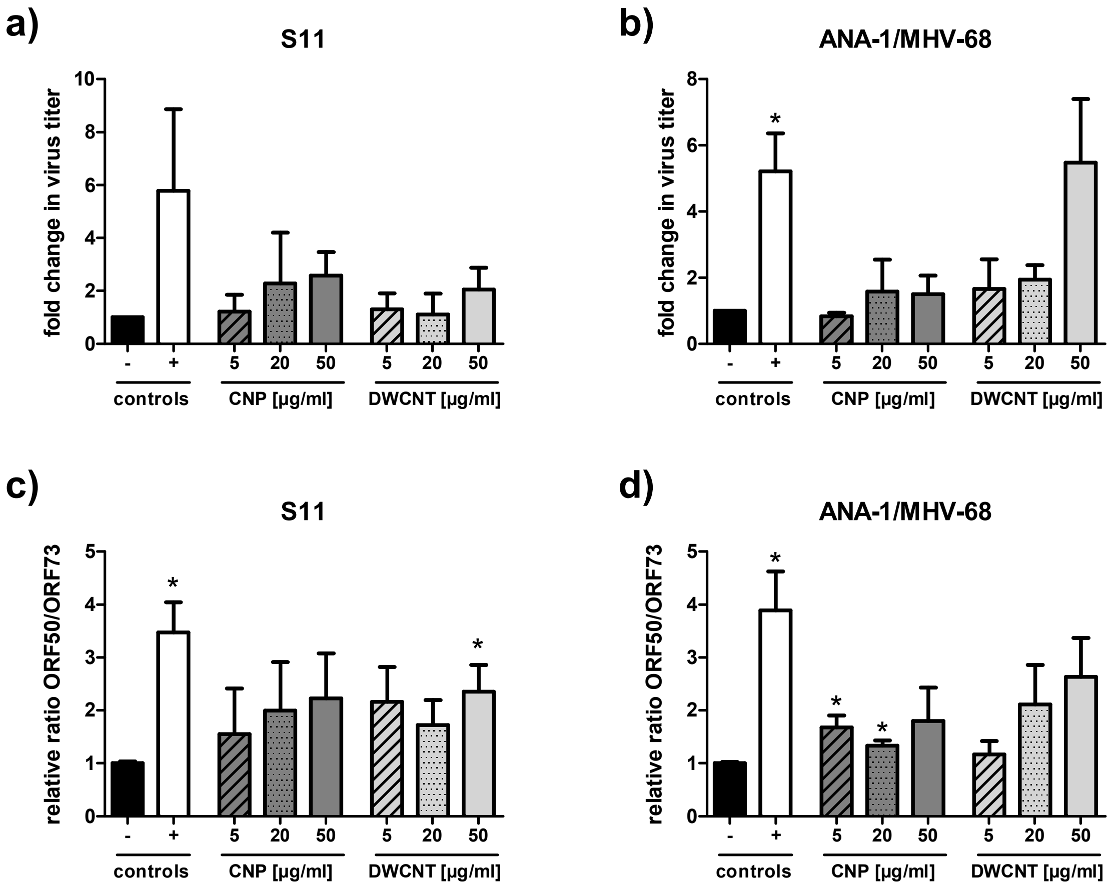

Figure S3. Exposure to NP reactivates lytic virus in persistently infected cells in vitro in a dose dependent manner: The persistently with MHV-68 infected cell lines S11 (B cell line) and ANA-1/MHV-68 (macrophage cell line) were incubated with 5 µg/ml, 20 µg/ml or 50 µg/ml NP. Treatment with TPA (in S11) or LPS (in ANA-1/MHV-68) was used as a positive control for virus reactivation. The amount of lytic virus in the supernatant was determined by plaque assay after 72 h (panels a and b). Expression of the viral genes ORF50 (specific for the lytic phase) and ORF73 (expressed during lytic and latent phase) – shown as the ratio ORF50/ORF73 – (panels c and d) was analyzed by RT-PCR 72 h after NP exposure. The values in untreated cells were set as "1" and the values for cells after NP treatment were calculated relative to the control. Data shown are the means + SD from three independent experiments. Asterisks indicate a statistically significant difference to the untreated control (*: $P < 0.05$).

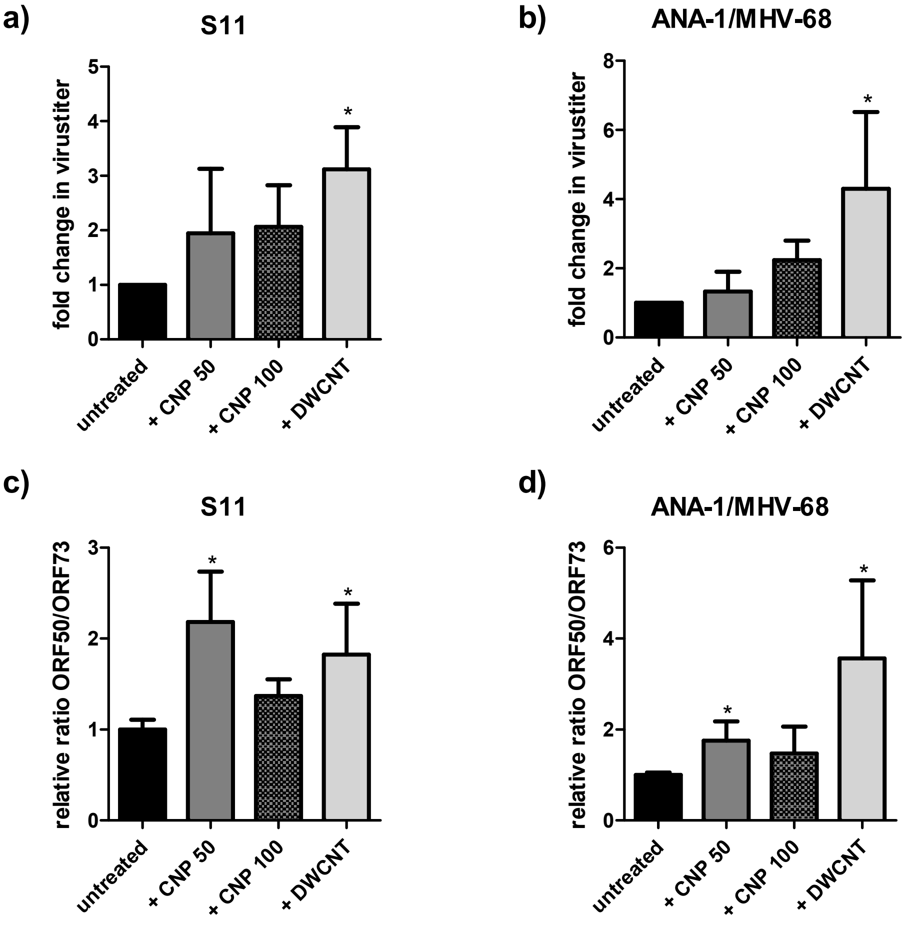

Figure S4. Exposure to NP reactivates lytic virus in persistently infected cells independently of the particle aspect ratio: To compare the effects of NP on virus reactivation adapted to their surface area, the persistently with MHV-68 infected cell lines S11 (B cell line) and ANA-1/MHV-68 (macrophage cell line) were incubated with 50 µg/ml CNP (total surface area according to BET equation: 136 cm^2/ml) or DWCNT (330 cm^2/ml) and 100 µg/ml CNP (272 cm^2/ml). The amount of lytic virus in the supernatant was determined by plaque assay after 72 h (panels a and b). Expression of the viral genes ORF50 and ORF73 – shown as the ratio ORF50/ORF73 – (panels c and d) was analyzed by RT-PCR 72 h after NP exposure. The values in untreated cells were set as "1" and the values for cells after NP treatment were calculated relative to the control. Data shown are the means + SD from three independent experiments. Asterisks indicate a statistically significant difference to the untreated control (*: $P < 0.05$).

a)

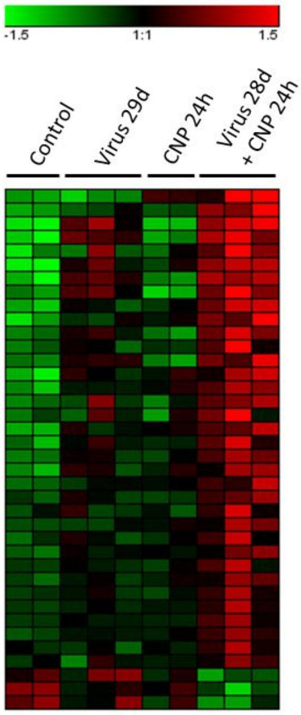

b)

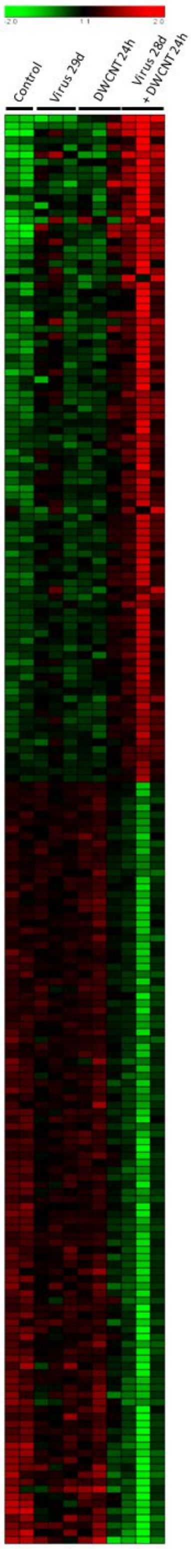

c)

Functions Annotation	p-value	z-score	activation state (# of genes)
proliferation of cells	1.10×10^{-8}	2.07	increased (106)
morbidity or mortality	5.44×10^{-8}	3.27	increased (80)
leukocyte migration	8.59×10^{-7}	2.70	increased (37)
immune response of cells	8.10×10^{-5}	2.32	increased (23)
activation of cells	3.19×10^{-4}	2.10	increased (32)

d)

Functions Annotation	p-value	z-score	activation state (# of genes)
organization of cytoskeleton	2.74×10^{-7}	-2.55	decreased (128)
microtubule dynamics	3.13×10^{-7}	-2.26	decreased (112)
proliferation of cells	9.15×10^{-7}	2.47	increased (277)
organization of cytoplasm	2.32×10^{-6}	-2.54	decreased (134)
tumorigenesis of tissue	2.09×10^{-3}	2.43	increased (615)

Figure S5. Short-time exposure of latently infected mice to NP differentially regulates gene expression in whole lung tissue: The transcriptome of whole lung tissue was analyzed with the Illumina-MouseRef-8v2.0 Expression BeadChip. Significantly altered genes with at least 1.5-fold increased or decreased expression values compared to all controls (non-treated mice and mice treated with MHV-68 for 28 days or NP for 24 hours alone) were considered as differentially regulated. Supplementary Figure 4a shows a heatmap of all regulated genes in the group subsequently treated with CNP and Supplementary Figure 4b shows a heatmap for genes regulated in the group treated with DWCNT. Cellular pathways found to be associated with the identified genes by IPA and their predicted activation states are shown in c) and d). Expression values of 3 mice per group are shown, except for the control group and the CNP 24h group where the expression values of 2 mice are shown.

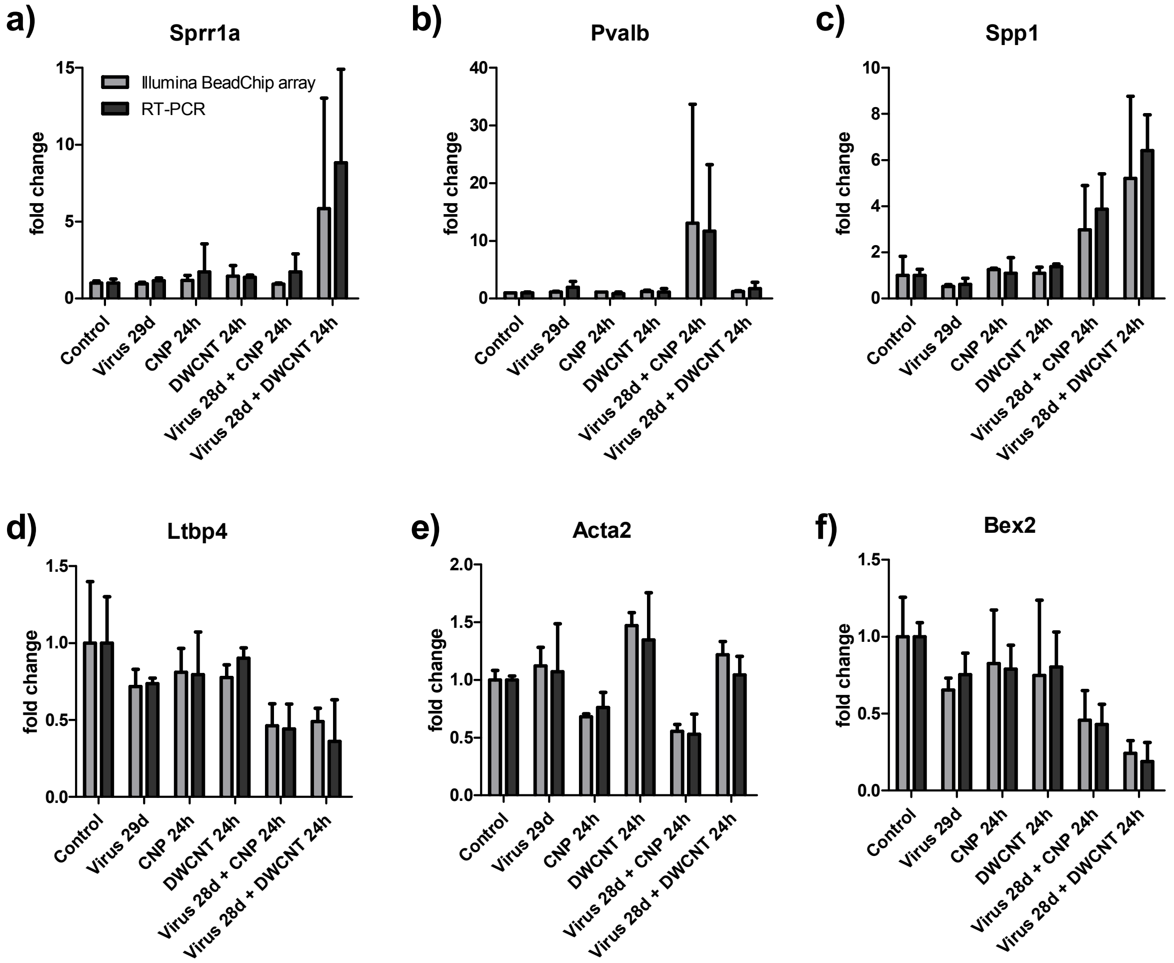

Figure S6. Confirmation of gene expression data by real-time quantitative PCR for selected genes: Genes that were found to be differentially expressed in the transcriptome analysis were also tested in samples from whole lung tissue by RT-PCR. 3 upregulated (a – c) and 3 downregulated genes (d – f) were chosen for analysis. The means + SD of 3 mice per group are shown, except for the array-data of the CNP 24h group where the mean of 2 mice is shown.

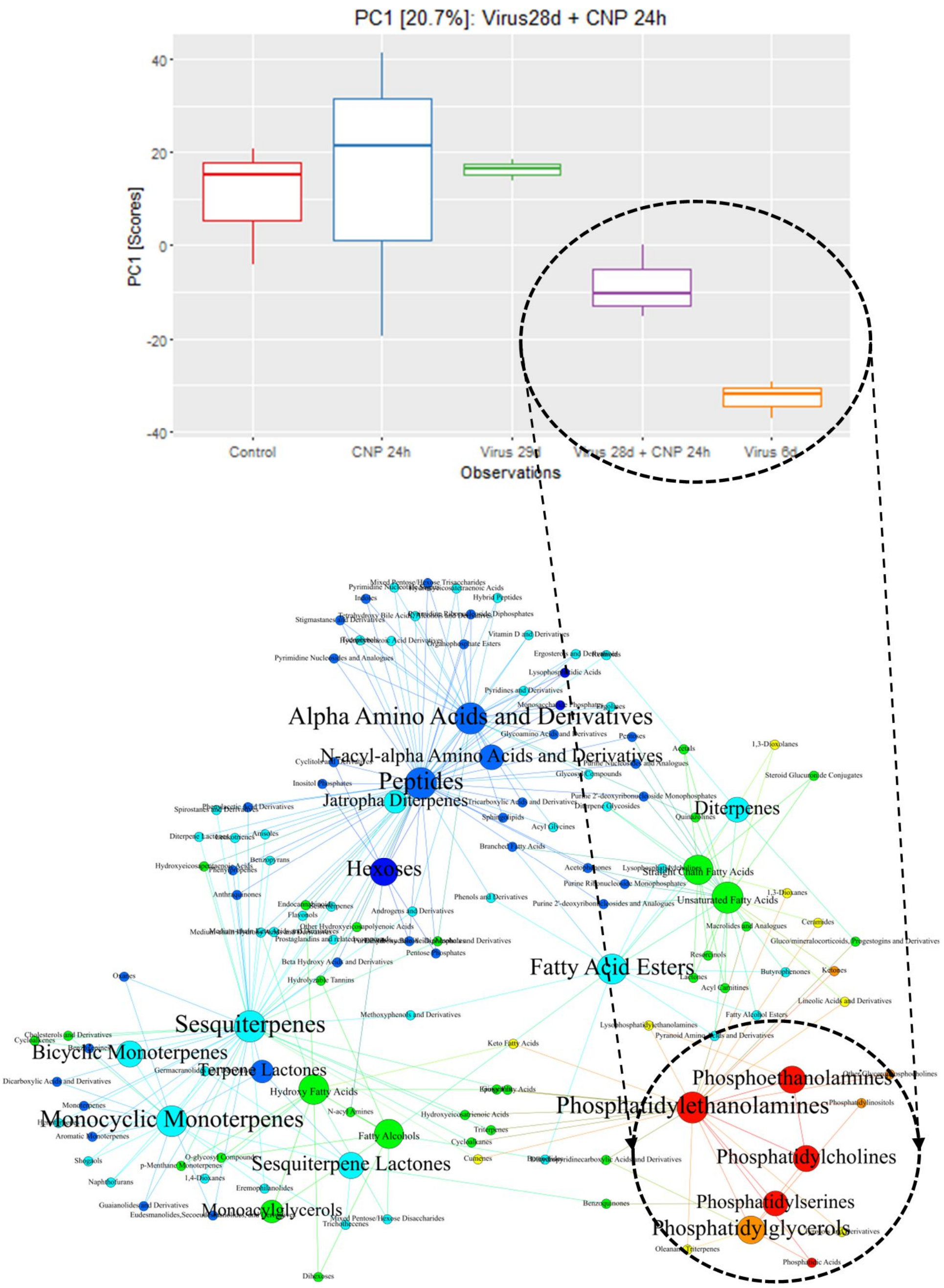

Figure S7. Exposure of latently infected mice to CNP leads to an increase in glycerophospholipids: Detailed analysis of the compound class pattern represented by the boxplot that was already shown in Figure 6a demonstrated upregulation of glycerophospholipids such as phosphoethanolamines, phosphatidylcholines, phosphatidylserines, and phosphatidylglycerols.

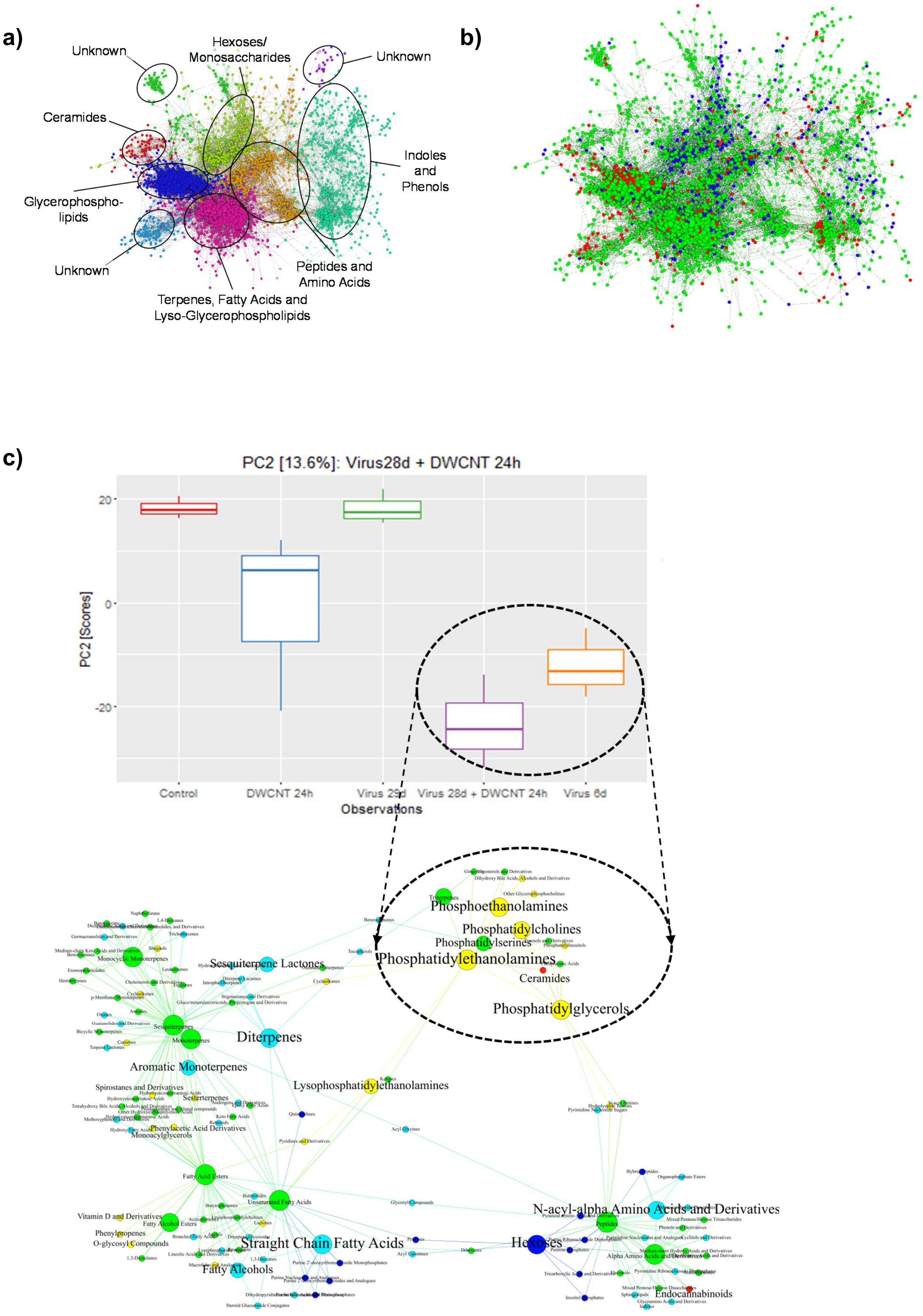
a)
Unknown
Hexoses/
Monosaccharides
Unknown
Ceramides
Indoles
and
Phenols
Glycerophospho-
lipids
Unknown
Peptides and
Amino Acids
Terpenes, Fatty Acids and
Lyso-Glycerophospholipids
b)
c)
PC2 [13.6%]: Virus28d + DWCNT 24h
PC2 [Scores]
20
0
-20
Control
DWCNT 24h
Virus 28d
Virus 28d + DWCNT 24h
Virus 6d
Observations
Phosphoethanolamines
Phosphatidylcholines
Phosphatidylserines
Phosphatidylethanolamines
Ceramides
Phosphatidylglycerols
Sesquiterpene Lactones
Diterpenes
Aromatic Monoterpenes
Lysophosphatidylethanolamines
Straight Chain Fatty Acids
Fatty Alcohols
N-acyl-alpha Amino Acids and Derivatives
Hexoses
Endocannabinoids

Figure S8. Exposure of latently infected mice to DWCNT leads to an increase in glycerophospholipids: Mass difference networks were created to visualize chemical similarities between latently infected mice that were exposed to DWCNT and acutely infected mice. Supplementary Figure 8a gives an overview of the compound classes that were detected. Panel b shows the mass difference network for latently infected mice treated with DWCNT, where green color stands for unchanged compounds. Blue color represents metabolites that were downregulated both in the group “Virus 28d+DWCNT 24h” and in “Virus 6d”. Red color depicts metabolites that were upregulated in both groups. Likewise, PCA showed a certain overlap between these groups (c) and detailed analysis of the involved compounds identified glycerophospholipids such as phosphatidylethanolamines and phosphatidylcholines as being upregulated.

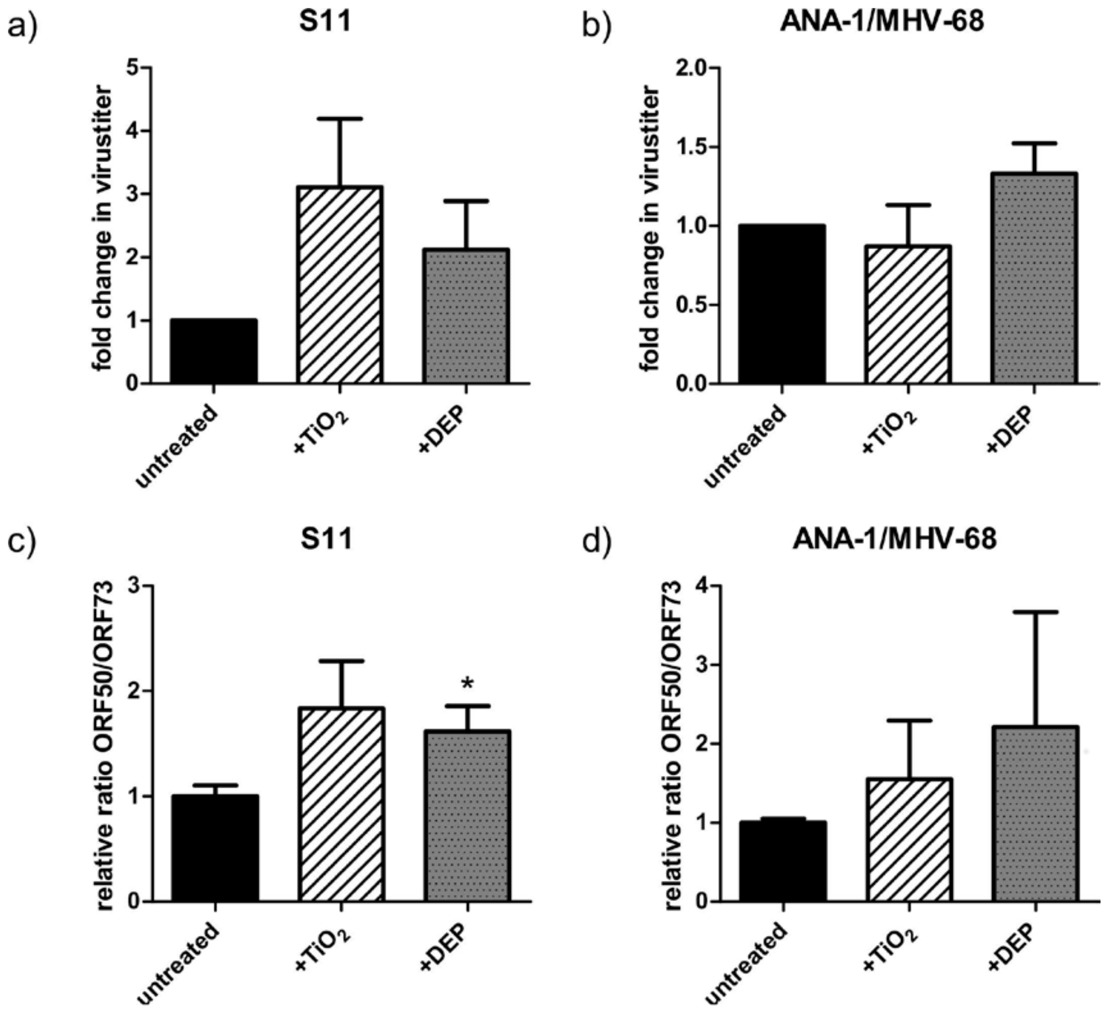

Figure S9. Exposure of persistently infected cells to TiO_2 NP or DEP has differential effects on virus reactivation in vitro: S11 and ANA-1/MHV-68 cells were incubated with 50 µg/ml TiO_2 NP or DEP and lytic virus was determined in the supernatant by plaque assay after 72 h (panels a and b). Expression of the viral genes ORF50 (specific for the lytic phase) and ORF73 (expressed during lytic and latent phase) – shown as the ratio ORF50/ORF73 - (panels c and d) was analyzed by RT-PCR 72 h after NP exposure in S11 and in ANA-1/MHV-68 cells. The value in untreated cells was set as "1" and the values for cells after NP treatment were calculated relative to the control. Data shown are the means + SD from three independent experiments. Asterisks indicate a statistically significant difference to the untreated control (*: $P < 0.05$).

Table S1: Gene expression values of selected genes.

		Expression values…						
**Gene**	**regulated by…**	**untreated**	**Virus 6d**	**Virus 29d**	**CNP 24h**	**Virus 28d + CNP 24h**	**DWCNT 24h**	**Virus 28d + DWCNT 24h**
Retnla	CNP, DWCNT	238 ± 23	6086 ± 869	512 ± 154	3574 ± 613	3386 ± 1693	7777 ± 3131	5842 ± 787
Cxcl1	CNP, DWCNT	76 ± 10	324 ± 94	93 ± 8	1012 ± 565	572 ± 195	362 ± 278	480 ± 130
Timp1	CNP, DWCNT	201 ± 6	1165 ± 251	273 ± 18	1438 ± 505	967 ± 463	2272 ± 2187	3214 ± 907
Slc26a4	CNP, DWCNT	59 ± 0	223 ± 77	98 ± 12	534 ± 266	339 ± 113	435 ± 259	239 ± 108
Lcn2	CNP, DWCNT	1197 ± 17	3149 ± 972	1432 ± 313	9092 ± 2956	5813 ± 1742	6178 ± 4072	6053 ± 1536
Spp1	CNP, DWCNT	112 ± 4	176 ± 58	109 ± 15	263 ± 8	621 ± 323	229 ± 44	1082 ± 602
Ch25h	CNP, DWCNT	111 ± 13	474 ± 150	138 ± 31	272 ± 94	241 ± 33	266 ± 145	440 ± 105
Serpina3n	CNP, DWCNT	651 ± 5	1179 ± 232	662 ± 46	1601 ± 347	1362 ± 350	2131 ± 861	2563 ± 689
BC048546	CNP, DWCNT	226 ± 48	649 ± 43	391 ± 139	661 ± 110	629 ± 88	768 ± 188	852 ± 173
Hmgcs2	CNP, DWCNT	460 ± 13	171 ± 26	403 ± 81	236 ± 60	210 ± 25	220 ± 90	126 ± 10
Saa3	CNP	49 ± 3	1354 ± 727	109 ± 29	2564 ± 898	3338 ± 2308	1564 ± 1885	2425 ± 1203
Ccl7	CNP	29 ± 2	328 ± 125	45 ± 2	79 ± 30	103 ± 32	77 ± 44	240 ± 149
Pigr	CNP	50 ± 2	183 ± 64	84 ± 17	139 ± 2	186 ± 77	145 ± 44	122 ± 36
LOC666904	CNP	3247 ± 24	6430 ± 1397	3345 ± 432	6480 ± 1757	5641 ± 491	4638 ± 1101	7140 ± 2031
Hpx	CNP	53 ± 2	79 ± 5	45 ± 3	100 ± 14	93 ± 23	99 ± 17	81 ± 26
Cd14	CNP	284 ± 19	527 ± 11	322 ± 41	757 ± 181	529 ± 76	463 ± 142	522 ± 98
Cxadr	CNP	520 ± 29	773 ± 147	489 ± 83	835 ± 93	721 ± 11	703 ± 93	495 ± 140
Aoc3	CNP	463 ± 95	204 ± 66	357 ± 138	131 ± 11	192 ± 63	270 ± 97	285 ± 150
Muc5ac	DWCNT	37 ± 3	112 ± 36	53 ± 11	61 ± 3	102 ± 58	108 ± 21	140 ± 67
Cp	DWCNT	376 ± 33	1314 ± 155	566 ± 27	785 ± 187	984 ± 289	1041 ± 192	1200 ± 29
Socs3	DWCNT	332 ± 3	751 ± 80	367 ± 12	744 ± 180	492 ± 21	613 ± 237	762 ± 23
Rrbp1	DWCNT	170 ± 17	278 ± 46	175 ± 13	290 ± 15	301 ± 86	254 ± 15	372 ± 94
Osmr	DWCNT	277 ± 9	682 ± 109	404 ± 29	622 ± 177	467 ± 16	617 ± 134	625 ± 57
Ppp1r14d	DWCNT	44 ± 0	105 ± 14	57 ± 1	89 ± 3	62 ± 2	94 ± 20	85 ± 24
Ctps	DWCNT	978 ± 93	1349 ± 139	795 ± 18	1730 ± 449	1156 ± 103	1332 ± 539	1636 ± 125
Cirbp	DWCNT	379 ± 6	206 ± 20	333 ± 52	210 ± 61	267 ± 34	213 ± 30	186 ± 33
Ccdc28b	DWCNT	202 ± 21	101 ± 17	204 ± 15	128 ± 32	157 ± 34	94 ± 24	106 ± 12

Table S1: Overview of expression values of selected genes that are regulated during acute virus infection, by the combination of latent virus plus NP and by NP alone as tested by Illumina-MouseRef-8v2.0 Expression BeadChip (means ± SD).
